# From immunological mechanisms to targeted therapies: a bibliometric analysis in the domain of research concerning neutrophil extracellular traps and pulmonary diseases (2006–2025)

**DOI:** 10.3389/fimmu.2026.1837492

**Published:** 2026-06-03

**Authors:** Yingqi Pan, Gang Liu, Hui Wang, Jie Zhu, Jiabing Tong, Zegeng Li, Qinjun Yang

**Affiliations:** 1 College of Integrated Traditional Chinese and Western Medicine, Anhui University of Chinese Medicine, Hefei, China; 2 College of Traditional Chinese Medicine, Anhui University of Chinese Medicine, Hefei, China; 3 Respiratory Medicine Department, The First Affiliated Hospital of Anhui University of Chinese Medicine, Hefei, China; 4 Anhui Province Key Laboratory of the Application and Transformation of Traditional Chinese Medicine in the Prevention and Treatment of Major Pulmonary Diseases, Hefei, China; 5 Institute of Respiratory Disease Prevention and Treatment of Traditional Chinese Medicine, Anhui Academy of Chinese Medicine Sciences, Hefei, China

**Keywords:** pulmonary diseases, neutrophil extracellular traps, bibliometrics, CiteSpace, VOSviewer

## Abstract

**Objectives:**

Neutrophil extracellular traps (NETs) are a crucial mechanism of the neutrophil immune response and exhibit complex dual pathological and physiological effects in pulmonary diseases. In recent years, this research domain has increased significantly, yet systematic bibliometric research remains absent. Therefore, this study utilizes bibliometric methods to thoroughly examine the research landscape and development sequence of this domain, aiming to provide theoretical references for future research.

**Methods:**

In this study, VOSviewer and CiteSpace were used to conduct a visualization analysis of the article concerning NETs and pulmonary diseases indexed in the Web of Science Core Collection (WoSCC) and Scopus databases from January 1st, 2006, to December 31st, 2025. Additionally, we utilized the R package bibliometric and Origin 2025b to optimize the data and visualization charts, ensuring that the analytical results were presented with greater clarity and intuitiveness.

**Results:**

This study covered 1, 539 articles from 561 journals. The results indicate that since 2006, this domain has seen a phased growth trend. The phase from 2010 to 2018 was a stable growth phase; After 2019, propelled by the COVID-19 virus infection (COVID-19), it entered a significant development phase, and reached its peak in 2022. In terms of influence, China and the United States are leading contributors. Mark R. Looney of the University of California is the core author, and “Frontiers in Immunology” is the most frequently cited journal. Through the visualization analysis of topic categories, keywords, and references indicates that research focal points in this domain have progressively transitioned from initial fundamental mechanisms and pathological descriptions to concerns regarding pulmonary inflammation, immune thrombosis, COVID-19, and biomarkers, subsequently extending to frontiers such as the NLRP3 inflammasome, interstitial lung disease (ILD), and tumor microenvironment (TME).

**Conclusions:**

This study is the first application of bibliometric methods to visually present research domains related to NETs and pulmonary diseases, revealing the correlation between NETs and ILD, lung cancer (LC), and respiratory infections, along with the underlying mechanisms, continues to be a hot topic in research. However, the predominant function of NETs in specific diseases and their potential utility as therapeutic targets still necessitate further systematic elaboration.

## 1 Introduction

Pulmonary diseases refer to a group of highly heterogeneous lesions where the affected areas involve the respiratory tract and lung parenchyma, resulting in abnormal respiratory function or damage to related tissue structures. It covers common and severe diseases such as chronic obstructive pulmonary disease (COPD), lower respiratory tract infection (LRTI), COVID-19 virus infection (COVID-19), cystic fibrosis (CF), interstitial lung disease (ILD), bronchial asthma (BA), and lung cancer (LC) (1). Such diseases generally exhibit clinical characteristics of high incidence rate, high recurrence rates, high disability rates, high mortality rates, and a tendency towards chronicity (2). Their overall severity ranks among the top globally. Taking COPD and LRTI as examples, they respectively rank fourth and fifth among global mortality causes, resulting in nearly 5.5 million deaths. With the acceleration of industrialization and modernization, as well as occupational exposure, air pollution, smoking, and passive smoking, there has led to a continuous rise in the incidence and mortality rates of pulmonary diseases, increasingly becoming major, common, and frequently occurring diseases that affect global public health (3, 4). Despite the continuous optimization of treatment strategies for various pulmonary diseases in recent years, including pharmacotherapy, oxygen therapy, pulmonary rehabilitation training, surgical interventions, and interventional treatments, which have improved the prognosis to some extent, the overall survival rate has not significantly improved.

Neutrophil extracellular traps (NETs) are extracellular network structures released by neutrophils in response to specific microenvironmental signals, mostly constituted of chromatin DNA, histones, myeloperoxidase (MPO), and neutrophil elastase (NE) (5). In pulmonary diseases, the formation and elimination of NETs exhibit significant dual pathological characteristics. On the one hand, in the initial phase of acute infection or inflammation, NETs effectively eliminate pathogens through physical capture and enzymatic degradation, playing a role in host defense. On the other hand, excessive or sustained NETosis can result in tissue injury, inflammation amplification, and immunologic derangement. Cytotoxic histones and active proteases carried by NETs can induce damage to alveolar epithelial cells, stimulate the release of pro-inflammatory cytokines IL-1β and IL-6, activate the endogenous coagulation pathway, and promote microvascular thrombosis and tissue fibrosis, thereby advancing the progression of various pulmonary diseases (1). Since the 21st century, with the successive emergence of landmark events such as the SARS epidemic, H1N1 influenza, and the COVID-19 pandemic, along with the implementation of pertinent policies by the World Health Organization (WHO), national health research institutes, and regional health organizations, the research into the correlation between NETs and pulmonary diseases, along with the development of associated pharmaceuticals, has emerged as a hot domain of research. In this context, the articles in this domain have exhibited a phased growth trend, with numerous original studies published that systematically elucidate the regulatory mechanisms of NETs in the pathological and physiological processes of pulmonary diseases, clarifying the significant application potential of targeted regulation of NETs in the treatment of pulmonary diseases and establishing a crucial theoretical foundation for the clinical translation of this domain. Consequently, it is essential to conduct a thorough review and analysis of the articles related to NETs and pulmonary diseases.

Bibliometrics is a research method that employs mathematical and statistical techniques for the quantitative examination of articles within certain research domains (6). In the domain of medical research, this method can systematically and objectively reveal the discipline’s development sequence, the distribution of core research, and the evolutionary trends of emerging frontiers, thereby effectively overcoming the subjective and fragmented limitations in traditional reviews and offering researchers an objective analytical framework. Therefore, this study adopts the bibliometric method to systematically organize and visually analyse the articles in the domain of NETs and pulmonary diseases, aiming to comprehensively present the knowledge structure of this domain, identify current research hotspots, and emerging frontiers. This is crucial for a comprehensive understanding of the pathological and physiological characteristics of NETs in pulmonary diseases and for developing new strategies for the prevention or treatment of various pulmonary diseases in the future (7).

## 2 Data and methods

### 2.1 Data sources and search strategy

This study obtained articles from the WoSCC and Scopus databases. WoSCC and Scopus, as globally acknowledged authoritative academic databases, have exceptional search accuracy and data consistency. They provide accurate screening, meticulous query optimization, and thorough result analysis, encompassing a wide range of academic publications. The combination of the two sets of data can significantly enhance the breadth and depth of bibliometric analysis, achieve complementary coverage of journals, effectively reduce disciplinary and regional biases, and increase the comprehensiveness of the data. This provides a reliable data foundation for academic evaluation and research hotspot analysis in the domain of NETs and pulmonary diseases (8–10).

The search method for this investigation was established as TS = ((“Extracellular Trap*” OR “Extracellular DNA Trap*”) AND (“Lung” OR “Pulmonary”)). Considering that the initial article in this domain was published in 2006, therefore, the search timeframe was established from January 1st, 2006, to December 31st, 2025. Simultaneously, to prevent discrepancies arising from database updates, all articles were searched and exported on December 31st, 2025, to ensure the data’s trustworthiness and timeliness. The detailed search approach is given in **Supplementary Table 1**.

### 2.2 Data screening and processing methods

A total of 2009 articles were searched in the WoSCC database, and 2445 in the Scopus database. To ensure the rigor and validity of the research, the following screening criteria were established: (i) The research topic is an article on the correlation between NETs and pulmonary diseases, without restrictions on the type of research (animal experiments, cell experiments, human sample studies); (ii) article types were limited to Article and Review article; (iii) the language of the articles was English. The following exclusion criteria were established: (i) article that only mentioned NETs or lung diseases but had low relevance to the topic; (ii) article lacking important information such as author details, journal name, and publication year; (iii) non-targeted article (Meeting Abstract, Editorial Material, Early Access, Letter, Note, Proceeding paper, Book Chapters, Correction, Short survey, Conference paper, etc.).

**Figure 1:**
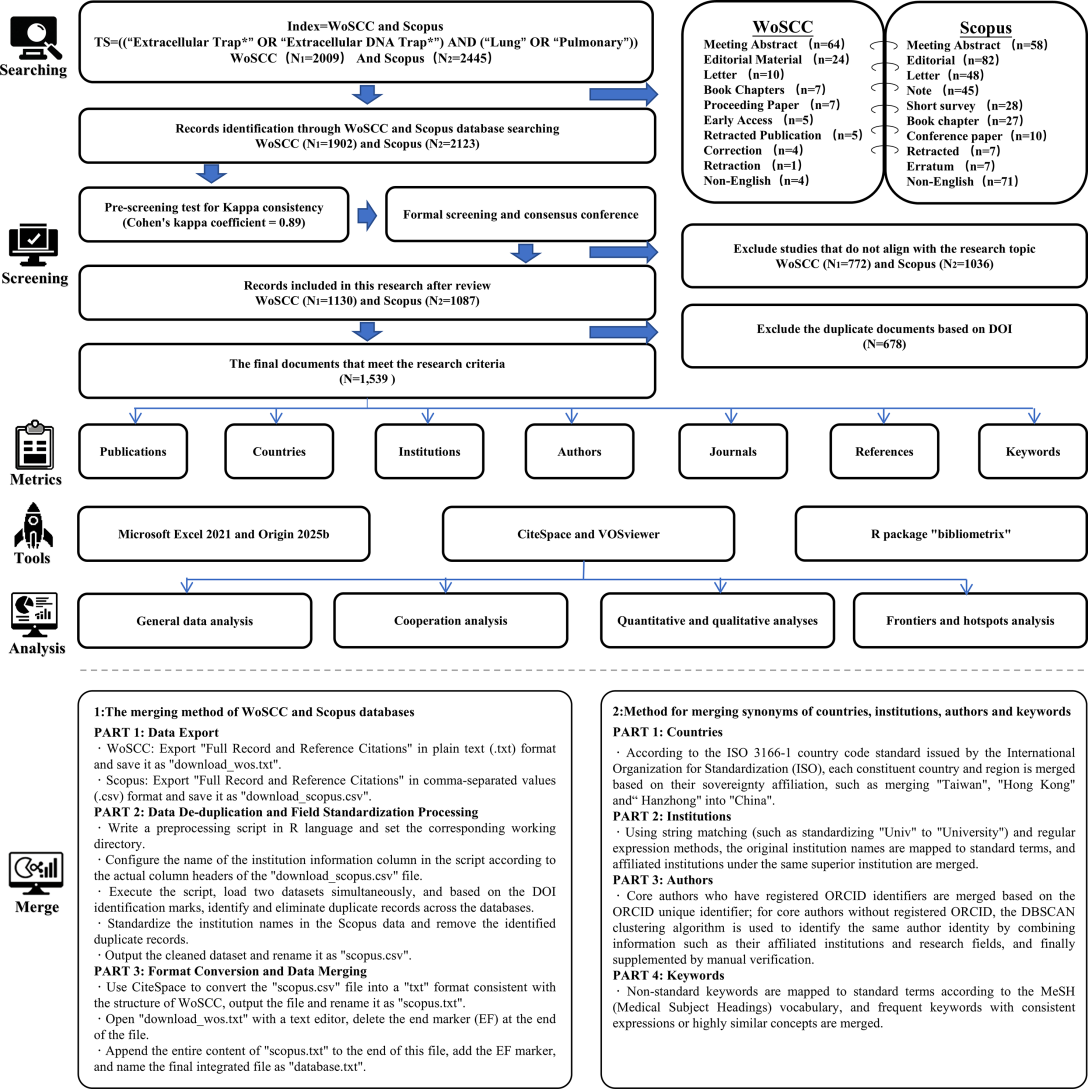
Article screening process and data merging method.

Before the formal screening process, two reviewers (YP and GL) conducted a pre-screening calibration using a randomly selected sample of 50 articles; the resulting Cohen’s kappa coefficient was calculated as 0.89, indicating an exceptionally high level of inter-rater agreement. Consequently, no modifications were made to the inclusion criteria or the review procedure. In the subsequent independent screening stage, all differences of opinion were resolved through consensus meetings, and the final decision was made by the senior author (QY). The article screening and verification work was completed on January 2nd, 2026, after standardizing the format, removing duplicates, and merging the data; a total of 1, 539 articles that met the criteria were ultimately included. The specific screening process and data merging methods are detailed in **Figure 1**.

### 2.3 Bibliometric analysis and visualization

This study employed tools such as CiteSpace (v6.4.R1), VOSviewer (v1.6.20), and the bibliometric package in R language (v4.5.1) to systematically present the results of bibliometric analysis and visualization.

CiteSpace is primarily used to identify the knowledge structure, hotspots, frontiers, and evolution dynamics in the research domain related to NETs and pulmonary diseases (11). This study primarily employs it to establish journal dual-map superimposition graphs, reference co-citation networks, and keyword timelines, and to identify references and keywords with high burstiness. The specific parameter settings are as follows: the time slice is one year, the node screening uses the g-index (k = 25) standard, and the network structure optimization employs the Pathfinder and Pruning the merged network algorithm. Among them, LRF = 2.5, L/N = 10, LBY = 5, e = 1.0. While maintaining the integrity of the network structure, redundant connections are effectively eliminated. The burst detection model uses the exponential distribution model f(x) = αe^−αx^ (with parameter α_1_/α_0_ = 2.0, α_i_/α_i−1_ = 2.0), and the maximum number of burst items = 35 to ensure the representativeness of the emergent nodes.

VOSviewer is primarily used to establish collaboration and co-citation networks to systematically reveal collaboration patterns and thematic structures within research areas (12). This study utilizes it to construct collaboration networks of countries, institutions, and authors, as well as co-occurrence networks of keywords. For the Co-authorship and Co-occurrence modules, set the minimum node occurrence frequency to 5 (with the minimum occurrence frequency for nodes in the keyword co-occurrence network set to 10), and balance the node spacing by optimizing attractive and repulsive forces (parameters attraction = 3, repulsion = -3) to improve the readability of the graph. The nodes on the visualization map represent countries/regions, institutions, authors, and keywords, respectively. Their sizes and hues show the frequency of occurrence as well as the cluster to which they belong, the length and thickness of the lines indicate the strength of the link, and the color-coded clustering modules clearly indicate the research domain’s thematic and structural qualities (13).

The R is primarily used to merge literature records from the WoSCC and Scopus databases, eliminate duplicate records, and standardize domain formats, thereby constructing a consistent and low-bias analytical dataset. Based on this dataset, the bibliometric package’s biblioAnalysis and H-index functions were employed to extract the dataset’s basic information, and the H-index (a comprehensive indicator for quantifying academic productivity and influence) of each country, institution, and author is then produced using the merged data, serving as a crucial metric for assessing academic output and comprehensive influence in this domain.

## 3 Results

### 3.1 Analysis of annual publication trends

**Figure 2:**
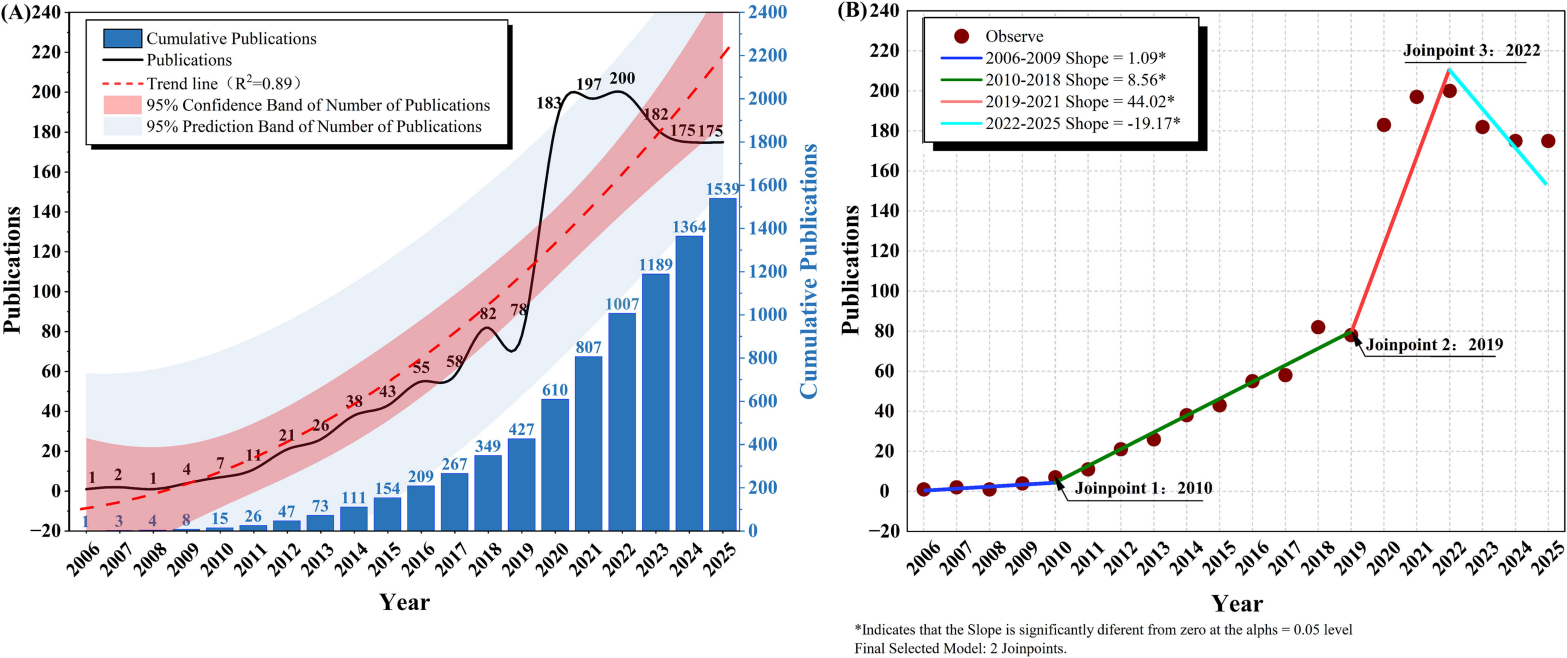
Distribution and trend analysis of publications in the domain related to NETs and pulmonary diseases. **(A)** Annual distribution chart of publication volume (the red dotted line represents the fitted trend line, the formula is y = 0.4922x² − 1972.08955x + 1975387.052, the shaded area represents the 95% confidence band and prediction band). **(B)** Publication output stage classification based on joinpoint regression analysis, the asterisk indicates that the slope of this stage is significantly different from zero at the α = 0.05 level. Final selected model: 3 joinpoints.

Performing an academic productivity analysis in the domain of NETs and pulmonary diseases can elucidate developmental dynamics and emerging trends in this domain. A total of 1, 539 publications were included in this study, originating from 2, 407 institutions across 68 countries/regions and authored by 9, 351 academics. They were published in 561 academic journals and were cited 85, 291 times in total. **Figure 2A** illustrates the trend in annual publications within this domain. The results indicate that the total publication has demonstrated a consistent upward trend since 2006 (with a fitted trend line exhibiting an R² value of 0.89, indicating a well-fitted model), in 2016, the publication volume surpassed 50 (n = 55, pct = 3.57%), increased to 183 in 2020 (pct = 11.89%), and peaked at 200 in 2022 (pct = 13.00%). The global COVID-19 pandemic appears to have boosted the rapid development of this domain. Additionally, this study identified 2010, 2019, and 2022 as important turning points in publication volume through the joinpoint regression analysis methodology. The results indicated that the growth slope was 1.09 from 2006 to 2009, 8.56 from 2010 to 2018, and 44.02 from 2019 to 2021. Notably, during the phase from 2022 to the data retrieval cut-off date, as the stimulating effect of the COVID-19 pandemic on scientific research output gradually subsided, the annual publication volume in this domain tended to stabilize, resulting in a slope of -19.17. Statistical analysis revealed that the variations among the four slopes were all statistically significant (p< 0.05), suggesting a significant shift in the growth trend at these turning points (**Figure 2B**).

Based on the publication volume and the growth slope, the academic achievements in this research domain can be categorized into three stages. Between 2006 and 2009, it was the initial exploration phase, with an average annual publication volume of less than 5, but demonstrating a consistent overall increase. Between 2010 and 2018, it was the stable growth phase, yielding a total of 341 relevant publications (pct = 22.16%) and a compound annual growth rate (CAGR) of 36.02%, indicating the persistent advancement of this research domain. After 2019, it entered a significant development phase, with publication volume rapidly increasing to 1, 190 (pct = 77.32%). Although the publication volume in 2025 was the same as that in 2024, the fitting findings of the current output and growth trends suggest that the intensity of academic activity in this domain will continue its upward trajectory.

### 3.2 Analysis of countries

**Figure 3:**
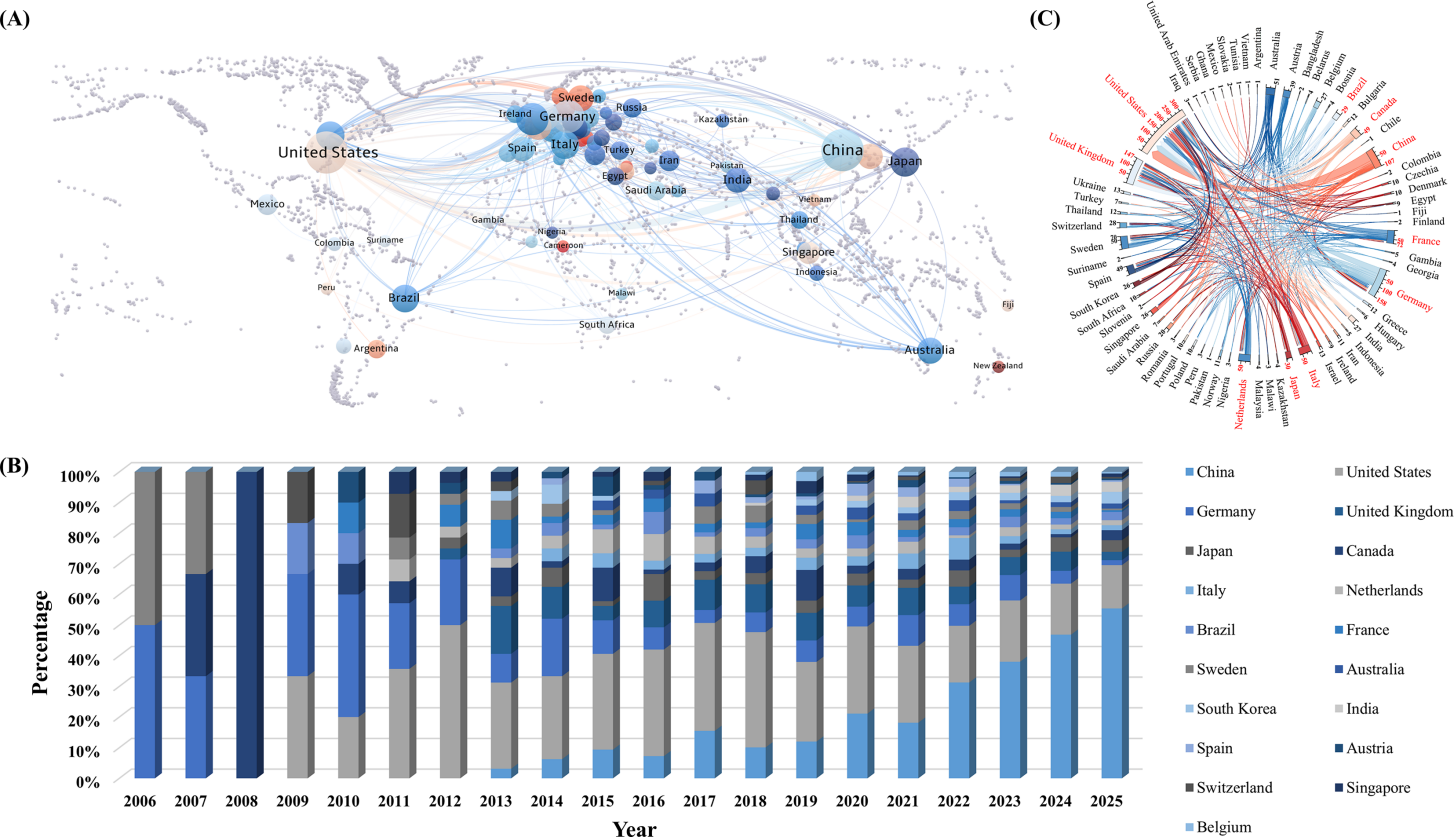
Academic output and cooperation situation of various countries in the domain related to NETs and pulmonary diseases. **(A)** Network visualization chart of countries. **(B)** Evolution chart of the annual proportion of academic output of each high-output country in this domain from 2006 to 2025. **(C)** International scientific research cooperation chord diagram.

An analysis of scientific research output and collaboration models at the national level can systematically elucidate the global development pattern, research differences among countries, and the structural characteristics of the collaboration network in the research domain related to NETs and pulmonary diseases. **Figure 3A** illustrates the global geographical distribution of countries in this domain and the extent of their collaboration. The results indicate that the international scientific research collaboration network is predominantly situated in the Northern Hemisphere, particularly in North America (n = 536, pct = 34.83%), Europe (n = 694, pct = 45.09%), and East Asia (n = 596, pct = 38.73%), which occupy preeminent roles in global academic output. Research in the Southern Hemisphere predominantly focuses on South America (n = 69, pct = 4.48%) and Oceania (n = 43, pct = 2.79%), with these regions maintaining strong connections to European and American countries. In contrast, the academic output and engagement of the African region (n = 21) are notably low, comprising merely 1.36% of the worldwide total, indicative of its peripheral status within the research network.

**Figure 3B** illustrates the differences of high-output countries (n≥20) over the research period in this domain. The results indicate that Germany and Sweden were the forerunners in commencing NETs-related research concerning pulmonary diseases, and lasted the longest. After the 2009 H1N1 influenza pandemic, countries such as the United States, Japan, and Brazil successively commenced relevant research. Notably, since China entered this domain in 2013, its research output has expanded significantly, and by 2025, its publication volume ranked first worldwide. Subsequently, propelled by the COVID-19 pandemic, this domain experienced a surge in academic output, marked by a notable rise in the number of participating nations, reflecting an overarching trend of globalization and diversification.

**Figure 3C** illustrates the collaborative links among countries within the global transnational collaboration network. The results indicate the United States has a core position in the transnational collaboration network, having had 315 close collaborative partnerships with 44 nations. Following closely are Germany (32/158), the United Kingdom (37/147), China (26/107), and France (21/72). Notably, the collaboration between China and the United States has reached as high as 54 times, indicating shared research objectives and a resource-sharing framework in this domain. Furthermore, there are high levels of collaboration within the European region, particularly among France, Germany, the Netherlands, Spain, and Sweden, which has markedly elevated the overall research quality of the region.

**Table 1** lists the top 10 countries based on publication volume. Among them, China (n = 486, pct = 31.58%) and the United States (n = 453, pct = 29.43%) were significantly higher than other countries, constituting the primary research entities in this domain. In terms of the H-index, the United States (H-index = 152), Germany (H-index = 95), and China (H-index = 92) rank among the top, indicating significant academic influence. In terms of betweenness centrality (BC), the United States (BC = 0.51), Germany (BC = 0.25), the United Kingdom (BC = 0.22), and Italy (BC = 0.11) constitute four essential hubs in the global collaboration network. Overall, the United States and Germany have emerged as the core contributing countries in this domain, owing to their high publication volume, high influence, and high centrality. Conversely, while China excels in publication volume, its average citation frequency per article (AC/P) and BC are comparatively low, indicating that its research endeavors remain predominantly domestically focused, with significant potential for enhancement in international collaboration and external academic influence. Notably, nine of the top ten countries by publication volume are also among the top ten in global GDP, suggesting a potential correlation between academic output and national economic strength.

**Table 1:** Top 10 countries with the highest academic output in the domain related to NETs and pulmonary diseases.

Rank	Country/Region	Output(N = 1539, n%)	AC/P	H-index	BC	2025GDP Rank
1	China	486(31.58%)	29.78	92	0.10	2
2	United States	453(29.43%)	85.21	152	0.51	1
3	Germany	143(9.29%)	109.85	95	0.25	3
4	United Kingdom	127(8.25%)	66.79	63	0.22	6
5	Japan	71(4.46%)	24.66	37	0.07	4
6	Canada	69(4.48%)	115.54	65	0.02	10
7	Italy	63(4.09%)	50.40	46	0.11	8
8	Netherlands	57(3.70%)	82.14	50	0.06	18
9	Brazil	54(3.51%)	56.50	44	0.01	9
10	France	53(3, 44%)	76.89	39	0.03	7

AC/P, Average citation frequency per article; BC, Betweenness Centrality; 2025GDP Rank, Ranking of the gross domestic product in the first half of 2025.

### 3.3 Analysis of institutions

**Figure 4:**
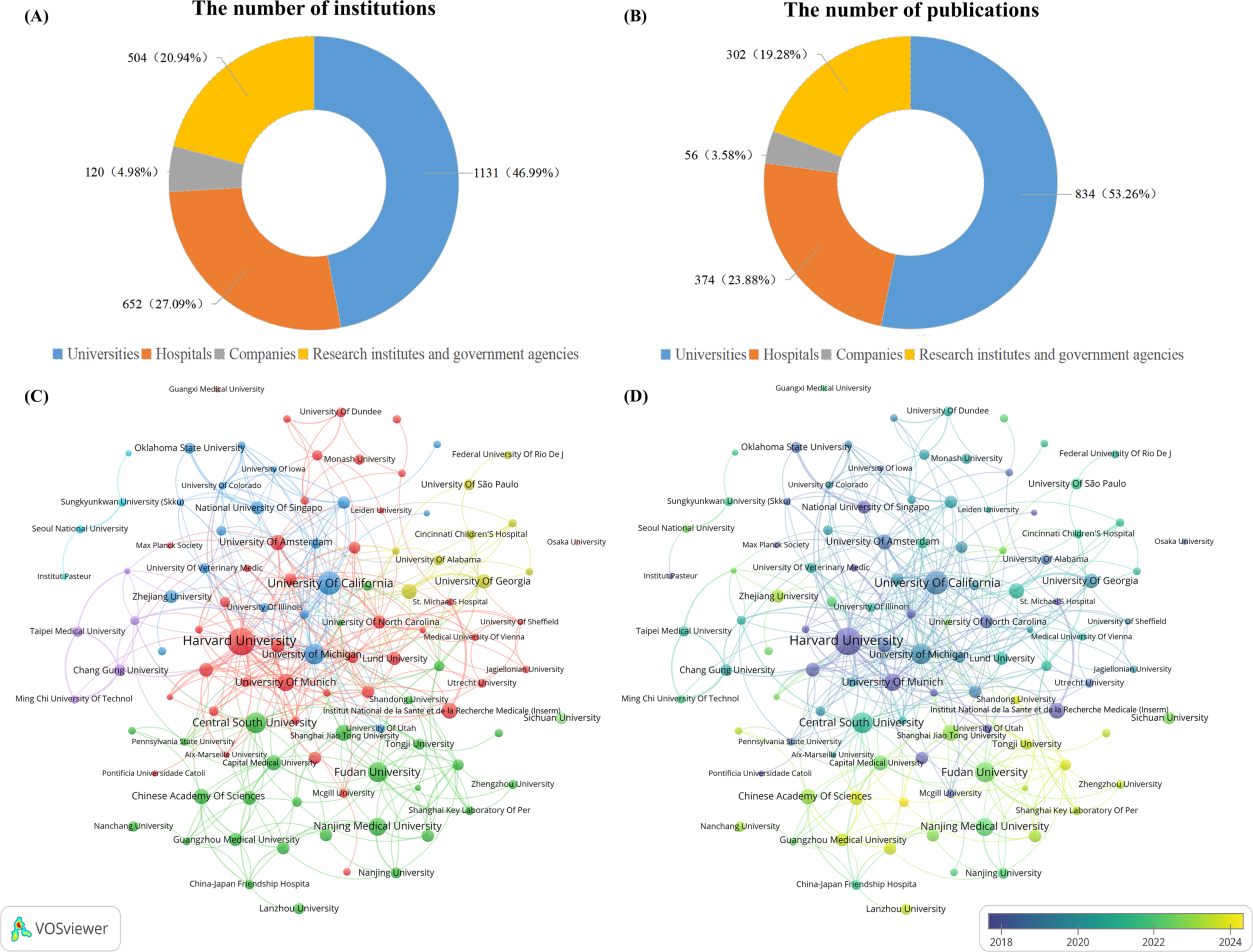
Institutional categories, outputs and cooperation situations in the domain related to NETs and pulmonary diseases. **(A)** Distribution chart of institutional categories. **(B)** Publication volumes of each category of institution. **(C)** Network chart of institutional cooperation. **(D)** Evolution chart of institutional cooperations over time.

Assessing the output and collaboration models of institutions helps elucidate the academic structure of NETs and pulmonary disease research domains. **Figure 4A, B** illustrate the distribution of institutional types and their corresponding academic output, among them, universities constitute the largest segment (n = 1131, pct = 46.99%), having published a total of 834 articles (pct = 53.26%); This is followed by research institutes and governmental agencies (n = 652, pct = 27.09%), hospitals (n = 504, pct = 20.94%) and companies (n = 120, pct = 4.98%). **Table 2** lists the top 10 institutions based on publication volume. The results indicate that American institutions, specifically Harvard University (n = 52, H-index = 48), the University of California (n = 40, H-index = 38), and the University of Michigan (n = 32, H-index = 29), excel in both publication quantity and influence, emphasizing the sustained scientific research competitiveness of the United States in this domain. Notably, although Chinese institutions have not yet attained a leading position in overall influence, they comprise half of the top 10 institutions by publication volume, including Central South University (n = 32, H-index = 17), Fudan University (n = 31, H-index = 17), Nanjing Medical University (n = 26, H-index = 25), Shanghai Jiao Tong University (n = 21, H-index = 17), and Capital Medical University (n = 20, H-index = 14), this reflects the rapid growth of research scale and a significant enhancement in overall research productivity in China within this domain.

**Table 2:** Top 10 institutions with the highest academic output in the domain related to NETs and pulmonary diseases.

Rank	Institutions	Output	AC/P	H-index	BC	Countries/Regions
1	Harvard University	52	141.60	48	0.42	United States
2	University of California	40	200.31	38	0.12	United States
3	University of Michigan	32	130.20	29	0.04	United States
4	Central South University	32	37.93	17	0.05	China
5	Fudan University	31	36.45	17	0.02	China
6	Nanjing Medical University	26	13.00	25	0.01	China
7	Inserm	21	72.95	14	0.07	France
8	Shanghai Jiao Tong University	21	109.22	17	0.03	China
9	University of Amsterdam	20	79.86	13	0.03	Netherlands
10	Capital Medical University	20	19.70	14	0.08	China

AC/P, Average citation frequency per article; BC, Betweenness Centrality; Inserm, Institut national de la santé et de la recherche médicale.

This study utilized the Co-authorship-Organizations module in VOSviewer to deeply analyse the collaborative network of institutions, identifying 111 institutions with a publication volume of five or more in this domain and establishing a collaboration network. The results indicate that only Harvard University (BC = 0.42) and the University of California (BC = 0.12) show comparatively high BC, signifying their roles as central output entities within their collaboration clusters and as essential hubs linking diverse regions and research domains. Notably, Chinese universities, including Central South University, Fudan University, Nanjing Medical University, and Shanghai Jiao Tong University, have established a localized cluster characterized by close collaboration and a stable framework, simultaneously, they are progressively assimilating into the international collaboration network via connections with prominent global institutions such as Harvard University and the University of California, thereby highlighting the sustained growth of Chinese institutions’ influence and engagement in this domain (**Figure 4C**). Further combining the institutional collaboration superimposed visualization graph reveals that the aforementioned institutions within the bluish-purple cluster are recognized as pioneers in collaborative research. In contrast, the majority of Chinese institutions are grouped in the yellow-green category, indicating that their international collaboration efforts have only gained relative momentum since 2022 (**Figure 4D**). Considering the publishing volume, AC/P, BC, and historical contributions, Harvard University is an established institution in this domain. Nevertheless, Chinese institutions exhibit significant potential for advancement due to their substantial pace of publication volume; they should enhance substantial collaboration with leading Western academic teams in the future.

### 3.4 Analysis of authors

Analysing author-level scientific output and collaboration networks can efficiently identify core authors and reveal essential information, including internal academic collaboration trends. According to Price’s Law, in a given research domain, the number of authors responsible for half of the publication is roughly equivalent to the square root of the total number of authors (**Equation 1**) (14). The minimum publication threshold m for core authors is determined by **Equation 2**, whereby n(x) represents the count of authors who have written x articles, n represents the total number of authors, and i = n_max_ reflects the publication volume of the most prolific author in the domain. In the domain of research concerning NETs and pulmonary diseases, the total author count n is 9, 351, and index i = n_max_ is 16; the minimal publication criterion m for core authors is calculated to be 3. Based on this, the research confirms that there are 97 authors in this domain who satisfy the core author criteria.

(1) $$\sum_{\text{m}+1}^{\text{I}}\text{n}(\text{x})=\sqrt{\text{n}}$$

(2) $$\text{m}=0.749\times \sqrt{{\text{n}}_{\max}}$$

According to **Table 3**, the top 10 core authors in terms of publication volume in this domain show significant heterogeneity in academic output and influence. The principal author, Rada Balazs (n = 16, H-index = 8), has substantial research productivity, yet his AC/P is comparatively low at 31.19, indicating that despite a prolific output, the influence of each article is restricted. In contrast, Looney, Mark R. (n = 12, H-index = 11) has accumulated 2, 759 citations, with an AC/P as high as 229.92, demonstrating excellent research quality and establishing himself as the preeminent core author in this domain. Moreover, certain authors such as Chalmers, James D. (n = 12, H-index = 10, AC/P = 63.00), Zhang, Hao (n = 10, H-index = 10, AC/P = 55.90), and Miao, Chang-Hong (n = 8, H-index = 6, AC/P = 68.63), despite commencing their research relatively recently (PY-start = 2021), have attained notable academic achievements, reflecting the rise and high activity of emerging researchers in this domain.

**Table 3:** Top 10 core authors with the highest academic output in the domain related to NETs and pulmonary diseases.

Rank	Author	Output	Citations	AC/P	H-index	PY-start
1	Rada, Balazs	16	499	31.19	8	2014
2	Mackman, Nigel	12	1157	96.42	12	2020
3	Looney, Mark R.	12	2759	229.92	11	2020
4	Chalmers, James D.	12	756	63.00	10	2021
5	Hwang, Tsong-Long	11	450	45.00	10	2020
6	Zhang, Hao	10	559	55.90	10	2021
7	Palaniyar, Nades	9	710	78.89	9	2015
8	Zhang, Si-Gong	9	149	16.56	4	2024
9	Miao, Chang-Hong	8	549	68.63	6	2021
10	Li, Hai-Tao	8	611	76.38	8	2019

AC/P, Average citation frequency per article; PY-start, Year of study initiation.

**Figure 5:**
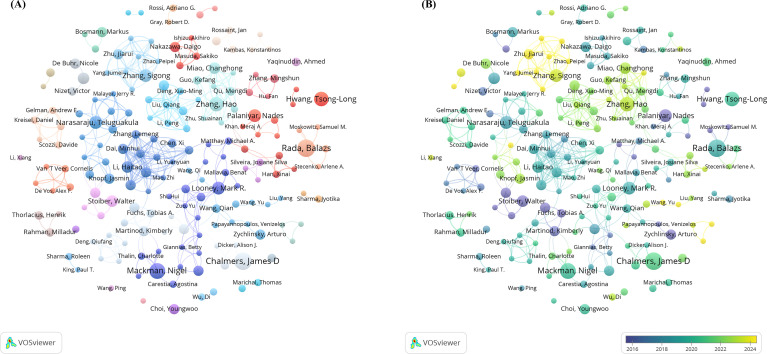
Cooperation situation of core authors in the domain related to NETs and pulmonary diseases. **(A)** Cooperation network chart of core author. **(B)** Evolution chart of core author cooperations over time.

**Figure 5A** illustrates the collaborative network among the authors. The results indicate that the collaboration network, concentrated on Chinese scholars such as Zhang Hao, Zhang Sigong, and Li Hai-Tao, has significant connectivity, forming a stable team with internal collaboration among multiple researchers. In contrast, the European and American authors represented by Mackman Nigel and Looney Mark R. have established a more extensive collaboration sub-network; however, their overall structure is more dispersed, exhibiting numerous relatively independent small collaboration clusters with distinct network clustering attributes. Moreover, certain authors, such as Bosmann Markus (Germany), Gelman Andrew E. (United Kingdom), and Rada Balazs (Hungary), exhibit a pronounced inclination for localized collaboration, characterized by relatively restricted cross-national or cross-institutional partnerships. The collaboration network has various phased properties from a temporal evolution standpoint. Initially, there were only a limited number of dispersed collaborative relationships, exemplified by the small-scale high-yield cluster established by Rada, Balazs, and Palaniyar, Nades, who can be considered the forerunners of the cooperation network in this domain. Since 2020, the extent of collaboration and network density has markedly escalated, peaking between 2022 and 2024. Overall, the collaboration model in this domain has progressively transitioned from an initial local and fragmented state to a network configuration characterized by multiple core teams serving as hubs, a considerable scale, and an increasingly complex structure (**Figure 5B**).

### 3.5 Analysis of journals

Identifying high-impact journals and knowledge diffusion paths within the domain can elucidate release trends and the disciplinary foundations of academic outputs. **Table 4** lists the top 10 journals ranked by publication volume and citation frequency. In terms of publication volume, “Frontiers in Immunology” (IF = 5.9) excels others with 112 articles, followed by “International Journal of Molecular Sciences” (n = 57, IF = 4.9), “Plos One” (n = 37, IF = 2.6), and “International Immunopharmacology” (n = 31, IF = 4.7), this distribution indicates that research achievements in this domain are predominantly focused in high-influence journals pertaining to immunology and its transdisciplinary domains. Notably, seven of the top 10 journals in terms of publication volume are classified in the JCR Q1 category, with four possessing an impact factor of more than 5. In terms of citation frequency, “Frontiers in Immunology” (n = 4792) ranks highest, followed by “Journal of Experimental Medicine” (n = 3789) and “Journal of Clinical Investigation” (n = 3740), indicating that these journals play a pivotal leading and supportive role in knowledge dissemination. Notably, eight of the top 10 journals by citation frequency are classified in the JCR Q1 category, and eight possess an impact factor of more than 5. Overall, “Frontiers in Immunology, “ “Plos One, “ “Journal of Thrombosis and Haemostasis, “ “Journal of Immunology, “ and “Journal of Experimental Medicine” are distinguished by their substantial publication volume and citation frequency, reflecting their considerable academic influence in this domain.

**Table 4:** Top 10 journals with the highest academic output and citation frequency in the domain related to NETs and pulmonary diseases.

Rank	Citing journals	Count	IF JCR	Cited journals	Citation	IF JCR
1	Frontiers in Immunology	112	5.9 Q1	Frontiers in Immunology	4792	5.9 Q1
2	International Journal of Molecular Sciences	57	4.9Q1	Journal of Experimental Medicine	3789	10.6Q1
3	Plos One	37	2.6Q2	Journal of Clinical Investigation	3740	13.6Q1
4	International Immunopharmacology	31	4.7Q1	Blood	2896	23.1Q1
5	Scientific Reports	28	3.9Q2	Plos One	2878	2.6 Q2
6	Respiratory Research	23	5 Q1	Journal of Immunology	2257	3.4 Q2
7	American Journal of Physiology-Lung Cellular and Molecular Physiology	19	3.5Q1	JCI Insight	2173	6.1 Q1
8	Journal of Thrombosis and Haemostasis	19	5 Q1	Journal of Thrombosis and Haemostasis	1527	5 Q1
9	Journal of Immunology	18	3.4Q2	Proceedings of the National Academy of Sciences of the United States	1385	9.1 Q1
10	Journal of Experimental Medicine	17	10.6Q1	American Journal of Respiratory and Critical Care Medicine	1319	19.4 Q1

**Figure 6:**
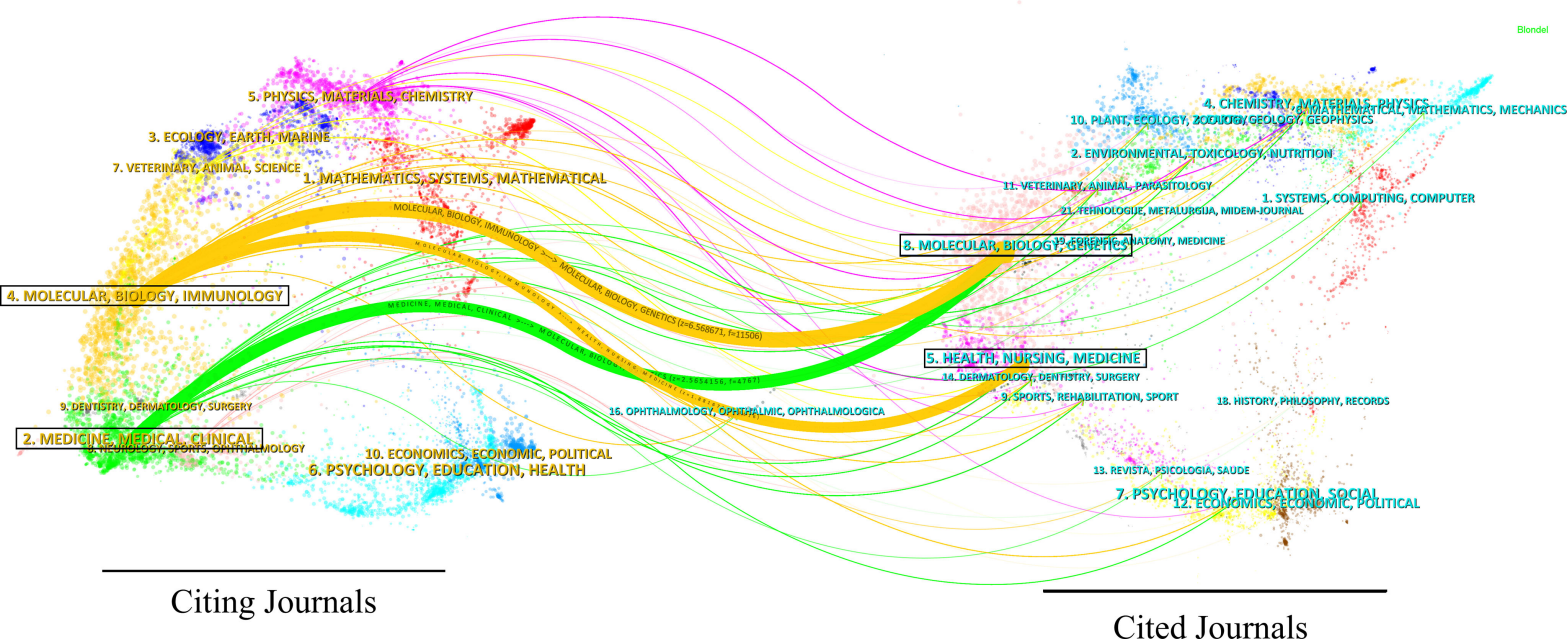
Dual- chart overlay analysis of interdisciplinary knowledge flow.

**Figure 6** illustrates the progression of knowledge citation and co-citation dynamics between citing and cited journals, elucidating alterations in topic distribution, citation patterns, and the trend of research focal shifts across academic journals (15, 16). The left side of the figure illustrates the subject distribution of the citing journals, representing the contemporary frontier directions of research domains; the right side illustrates the subject background of the cited journals, reflecting the theoretical foundation of the current research domains. The colorful curves in the figure illustrate the citation pathways among various journals, with the thickness of the pathways positively associated with the Z-score; a higher Z-score signifies a stronger citation relationship between the journals. The results indicate that research findings published in journals of the “MOLECULAR, BIOLOGY, GENETICS” and “HEALTH, NURSING, MEDICINE” domains are often cited by research in journals of the “MOLECULAR, BIOLOGY, IMMUNOLOGY” domain; furthermore, findings in the “MOLECULAR, BIOLOGY, GENETICS” domain are also referenced by research in the “MEDICINE, MEDICAL, CLINICAL” domain. This phenomenon highlights the ongoing evolution of fundamental molecular biology and genetics research towards immunology and clinical medicine in this domain, epitomizing a significant level of knowledge integration and the enhancement of interdisciplinary research. It elucidates that translational research has emerged as a vital path for the contemporary advancement of this subject.

### 3.6 Analysis of references

Analysing the highly cited articles within a research domain can elucidate the discipline’s development path, progressing from basic mechanistic analysis to detailed pathological and physiological exploration, and ultimately culminating in clinical research transformation. **Table 5** lists the 10 articles with the highest total citation frequency. Among them, the article “Neutrophil Extracellular Traps Kill Bacteria” by Brinkmann et al., published in Science in 2004, is the most cited, with 575 citations and an average of 26.14 citations per year, demonstrating substantial influence. This study primarily detailed the structural makeup of NETs and their antibacterial properties in experimental dysentery and spontaneous human appendicitis. Notably, this article was the initial publication to systematically elucidate NETs, establishing a new framework for researching the pathogenic mechanisms of pulmonary inflammation and infectious illnesses, and hence has maintained a high citation rate; Secondly, there are “Neutrophil Extracellular Traps in COVID-19” (Yu Zuo et al., published in JCI Insight in 2020) and “Neutrophil Extracellular Traps Contribute to immune thrombosis in COVID-19 Acute Respiratory Distress Syndrome” (Elizabeth A. Middleton et al., published in Blood in 2020). Despite the relatively low total citation frequency (TC) of these two articles (TC_1_ = 129, TC_2_ = 127), their annual citation frequency(ACF) exceeds 21 (ACF_1_ = 21.50, ACF_2_ = 21.17), suggesting that the levels of NETs in the serum of severe COVID-19 pandemic are markedly elevated and closely associated with inflammatory responses and thrombosis, this reveals that NETs could serve as a potential therapeutic target, this also reflects the swift response and significant focus of this research domain during a public health crisis. In contrast, earlier research by Axelle Caudrillier (2012) and Bárbara Nery Porto (2016), despite their citation frequencies ranking among the top ten, has exhibited a tendency to stabilize in influence with time (ACF_3_ = 4.36, ACF_4_ = 5.50).

**Table 5:** Top 10 most cited articles in the domain related to NETs and pulmonary diseases.

Rank	Title	Author	Journals	Year	TC	ACF
1	Neutrophil Extracellular Traps Kill Bacteria	Volker Brinkmann	Science	2004	575	26.14
2	Neutrophil extracellular traps in immunity and disease	Venizelos Papayannopoulos	Nature Reviews Immunology	2018	166	20.75
3	Neutrophil extracellular traps in COVID-19	Yu Zuo	Jci Insight	2020	129	21.50
4	Neutrophil extracellular traps contribute to immunothrombosis in COVID-19 acute respiratory distress syndrome	Elizabeth A Middleton	Blood	2020	127	21.17
5	Targeting potential drivers of COVID-19: Neutrophil extracellular traps	Betsy J. Barnes	Journal of Experimental Medicine	2020	118	19.67
6	SARS-CoV-2-triggered neutrophil extracellular traps mediate COVID-19 pathology	Flavio Protasio Veras	Journal of Experimental Medicine	2020	86	14.33
7	Maladaptive role of neutrophil extracellular traps in pathogen-induced lung injury	Emma Lefrançais	Jci Insight	2018	83	10.38
8	Platelets induce neutrophil extracellular traps in transfusion-related acute lung injury	Axelle Caudrillier	Journal of Clinical Investigation	2012	61	4.36
9	Neutrophil Extracellular Traps in Pulmonary Diseases: Too Much of a Good Thing?	Bárbara Nery Porto	Frontiers in Immunology	2016	55	5.50
10	Pulmonary Vascular Endothelialitis, Thrombosis, and Angiogenesis in Covid-19	Maximilian Ackermann	New England Journal of Medicine	2020	54	9.00

TC, Total citation count. ACF, Annual citation frequency.

**Figure 7:**
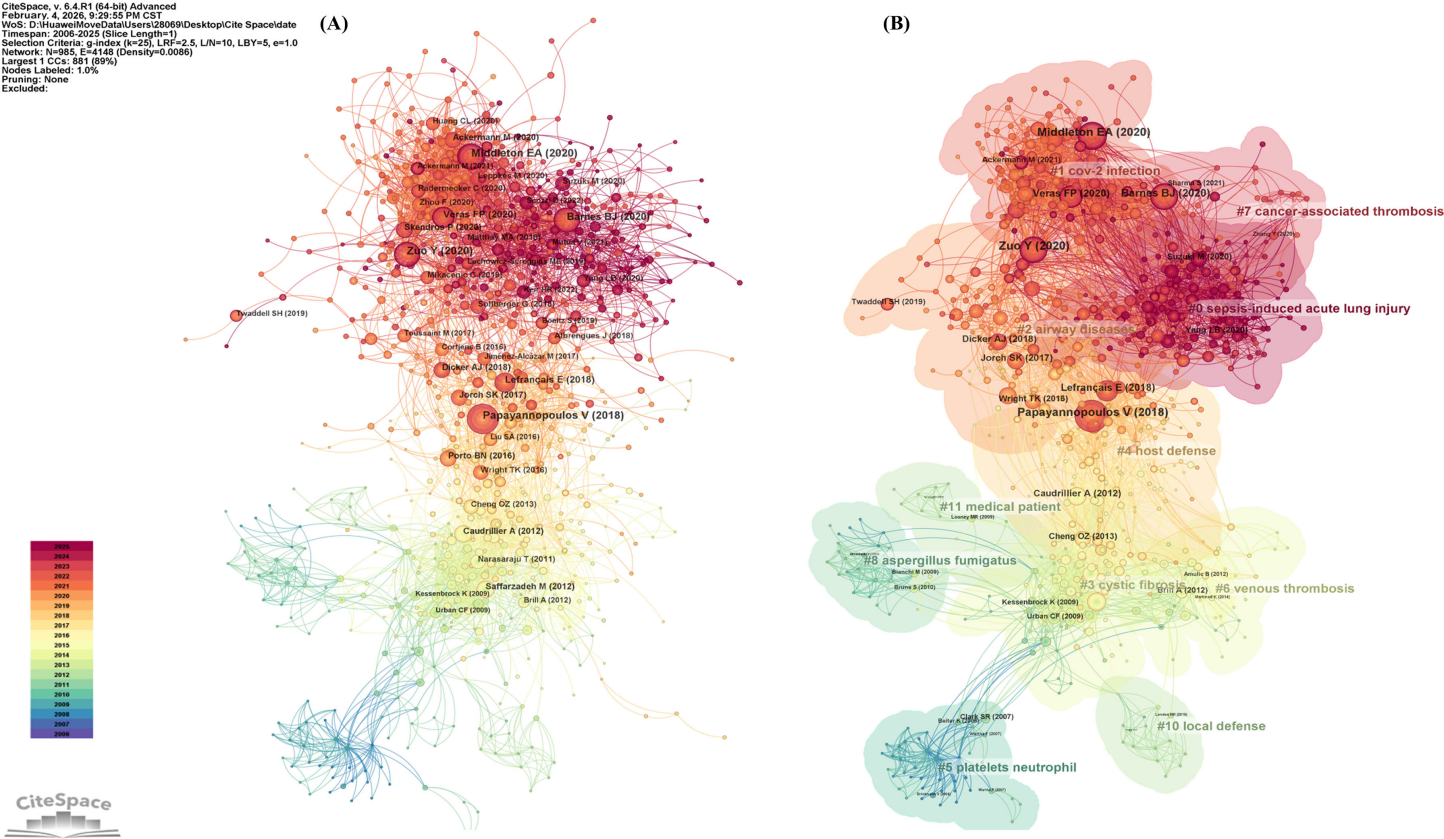
Reference literature analysis chart in the domain related to NETs and pulmonary diseases. **(A)** Co-occurrence network analysis chart. **(B)** Clustering network analysis chart.

**Figure 7A** illustrates the co-citation network of the references. Overall, the color of the nodes transitions from cool to warm tones, signifying the continual emergence of new references throughout time, which reflects the continuous accumulation and updating of knowledge in the research domain concerning NETs and pulmonary diseases. **Figure 7B** illustrates the results of the articles ‘ co-citation clustering analysis. Based on the strength of the connections between the articles, all articles are divided into 12 clusters, revealing the structural characteristics of knowledge evolution in this domain. Combining cluster scale and temporal evolution analysis, the origins of NETs and lung-related disease research can be traced back to five relatively autonomous research clusters: #3 (cystic fibrosis), #5 (platelets neutrophils), #8 (aspergillus fumigatus), #10 (local defense), and #11 (medical patient). These early clusters progressively fused and extended over time, resulting in more integrated research directions, such as #4 (host defense) and #2 (airway diseases). After 2019, the research focus on this domain became more concentrated and specialized, among them, three clusters #0 (sepsis-induced acute lung injury), #1 (cov-2 infection), and #7 (cancer-associated thrombosis) showed high correlation and intersection in content and methods, gradually evolving into more specialized and relatively independent frontier directions in current research. This clustering dynamic depicts the evolution of NETs in pulmonary diseases research, from dispersion to integration and finally to deepening in specialized domains.

**Figure 8:**
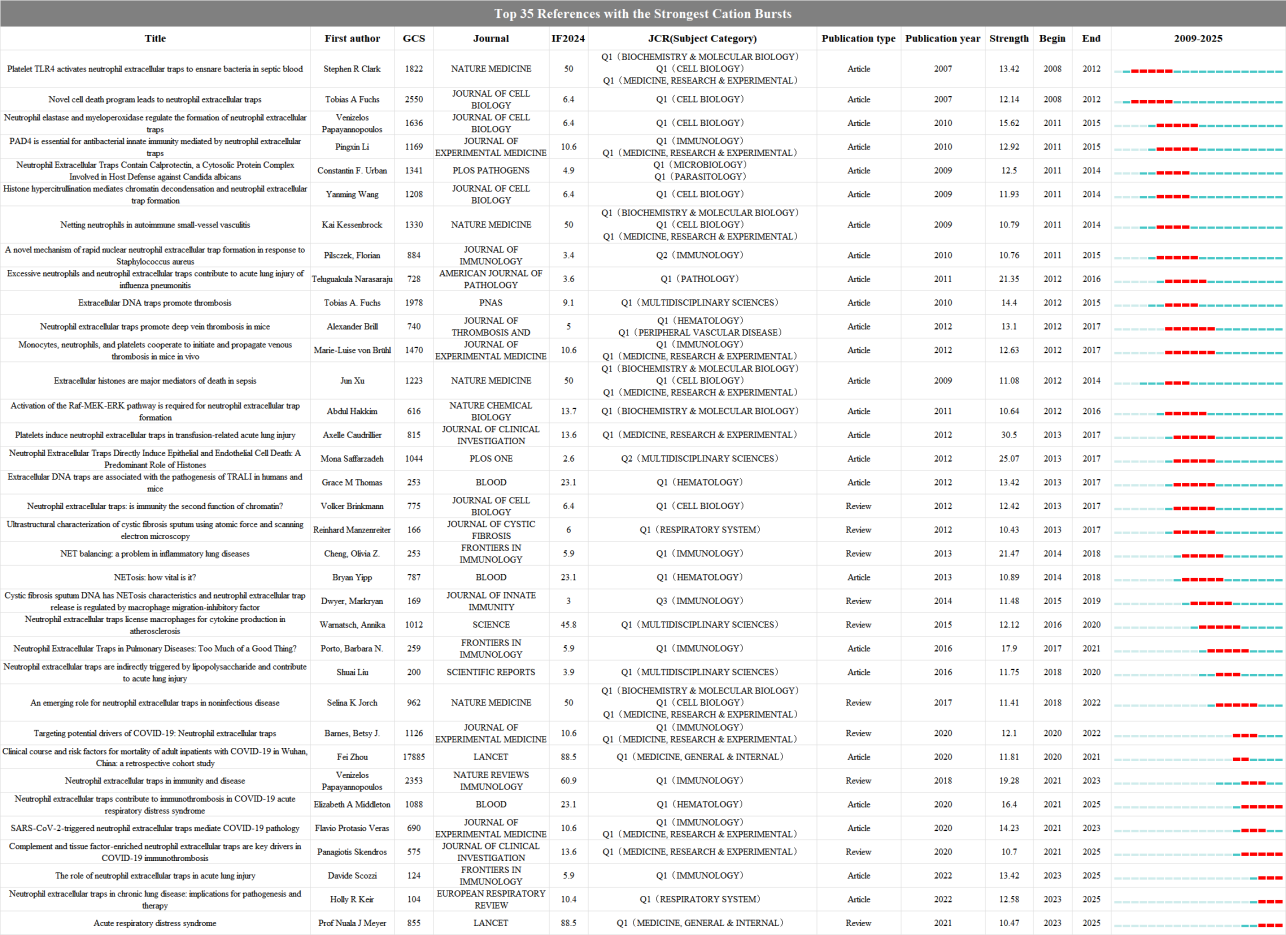
Top 35 references with the strongest citation bursts.

**Figure 8** illustrates the 35 references exhibiting the greatest burst intensity in this domain. The article “Platelets induce neutrophil extracellular traps in transfusion-related acute lung injury” by Axelle Caudrillier et al., published in the Journal of Clinical Investigation in 2012, ranks first with a burst intensity of 30.5, signifying its groundbreaking contribution to elucidating the primary mechanism of platelet-mediated NET formation in transfusion-related ALI (17). The subsequent article, “Neutrophil Extracellular Traps Directly Induce Epithelial and Endothelial Cell Death: A Predominant Role of Histones, “ authored by Mona Saffarzadeh et al. and published in PLOS ONE in the same year, exhibits a burst intensity of 25.07, the elevated burst value indicates the article’ substantial role in elucidating the direct cytotoxic mechanism of NETs, establishing a molecular basis for future pathological investigations into NETs-mediated tissue destruction (18). From the standpoint of temporal distribution in emergent articles, the citation bursts peaked in 2011(6 times) and 2012 (6 times), followed by 2013 (5 times), this indicates the articles from this phase predominantly discussed the formation mechanisms of NETs and their implications in pulmonary diseases, including ALI and pulmonary embolism, thereby promoting the initial wave of basic and translational research on NETs. Subsequently, there were four citation bursts in 2021, with the associated articles predominantly focusing on the role of NETs in infectious and immunological diseases such as COVID-19. As of 2023, five COVID-19-related articles have exhibited continuous citation surges (numbers 27, 28, 30, 31, and 32), addressing the correlation between NETs and immune thrombosis, cytokine storm, and lung tissue damage, indicating that the involvement of NETs in pulmonary diseases, particularly in the pathological mechanisms of COVID-19, remains a focal point of interest.

### 3.7 Analysis of keywords

**Figure 9:**
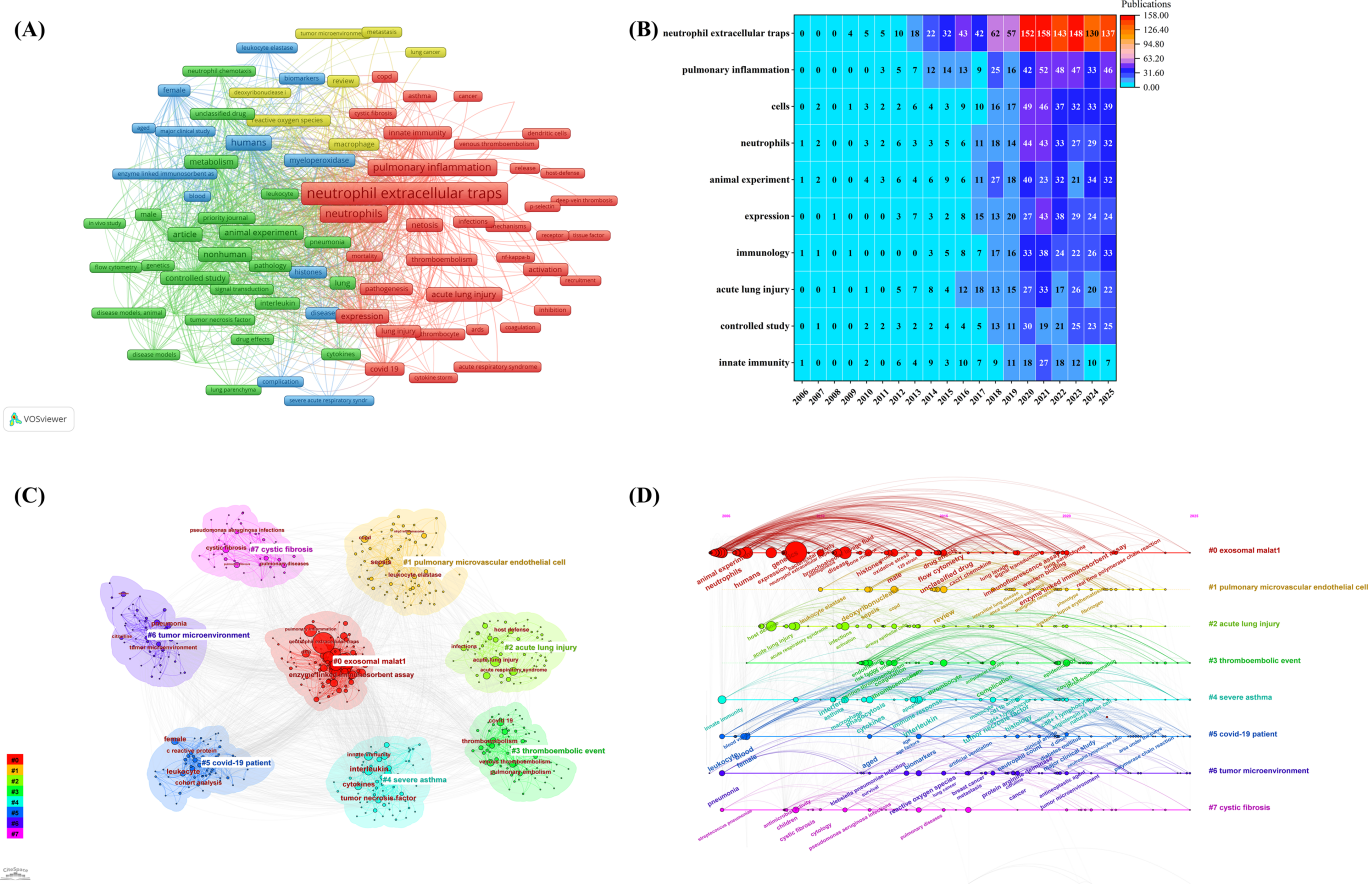
Keywords analysis in the domain related to NETs and pulmonary diseases. **(A)** Keyword co-occurrence chart. **(B)** Time heatmap of high frequency keywords. **(C)** Keyword co-occurrence clustering chart. **(D)** Keyword timeline chart.

Conducting keyword analysis is helpful for understanding the hot topics, frontier trends, and technological advancements in the research domain related to NETs and pulmonary diseases. After data cleaning, a total of 4, 215 keywords were obtained, with a cumulative occurrence of 26, 354 times. According to Price’s Law, keywords with an occurrence frequency of 43 or more were defined as high-frequency keywords in this study, totaling 85 keywords with a cumulative occurrence of 12, 956, accounting for 49.16% of the total frequency. **Figure 9A** illustrates the co-occurrence network of keywords. The results indicate that the three keywords with the highest occurrence frequencies are all closely related to the research domain, namely “NETs” (n = 1, 168, pct = 4.43%), “pulmonary inflammation” (n = 372, pct = 1.41%), and “neutrophil” (n = 282, pct = 1.07%). Following these are “acute lung injury” (n = 229, pct = 0.87%) and “innate immunity” (n = 154, pct = 0.58%). To deeply examine the temporal evolution of research hotspots, **Figure 9B** further illustrates the frequency distribution of high-frequency keywords over the years. Overall, the number of high-frequency keywords has increased gradually since 2016, and the majority of keywords have shown a continuous upward trend. By around 2020, the frequency of occurrence of high-frequency keywords has significantly increased, indicating that the research activity and attention in this domain have entered a phase of significant development. **Figure 9C** illustrates the clustering results of keywords. The results indicate that Modularity (Q) = 0.5401 (>0.3), Silhouette (S) = 0.8154 (>0.7), indicating that the keyword clustering structure in this domain is significantly and highly reliable, further analysis of the clustering scale reveals that the top 3 keywords in this domain are clustering (#0) exosomal malat1, clustering (#2) acute lung injury, and clustering (#4) severe asthma.

**Figure 9D** illustrates the timeline distribution of keywords related to the research domain of NETs and pulmonary diseases. The results indicate that before 2016, keywords such as “animal experiment”, “neutrophils”, “humans”, “innate immunity”, and “expression” frequently appeared, indicating that the research at that phase mainly focused on the expression characteristics of NETs in animal models and human tissues, and conducted basic explorations of its formation mechanism based on animal experiments and clinical samples. At the same time, the concentrated appearance of keywords such as “cystic fibrosis”, “thromboembolic event”, “asthma”, and “infections” indicates that the research focus during this phase was on the functional analysis of NETs in pulmonary disease models. After 2016, keywords such as “malignant neoplasm”, “COVID-19”, and “interstitial lung disease” became research hotspots, indicating that the domain gradually expanded from basic mechanism research to the construction of the role network of NETs in various pulmonary diseases, with particular emphasis on clarifying the complex correlations between NETs and the occurrence and development of ALI, and COVID-19, etc. At the same time, the frequency of occurrence of keywords such as “ western blotting”, “ enzyme-linked immunosorbent assay”, and “tumor microenvironment” significantly increased, suggesting that the research hotspots have further extended to the precise regulatory mechanisms of NETs in pulmonary diseases and their potential roles in cross-pathological backgrounds. This evolution trend not only reflects the systematic and diversified understanding of the mechanism of NETs, but also indicates that the domain is gradually shifting from basic mechanism exploration to application research in multiple disease scenarios and the development of targeted intervention strategies, in particular, the emergence of cutting-edge directions such as the regulatory mechanism mediated by exosomes, immunotherapy combination strategies, etc, has laid the theoretical and technical foundation for NETs as a new target for pulmonary disease therapy.

**Figure 10:**
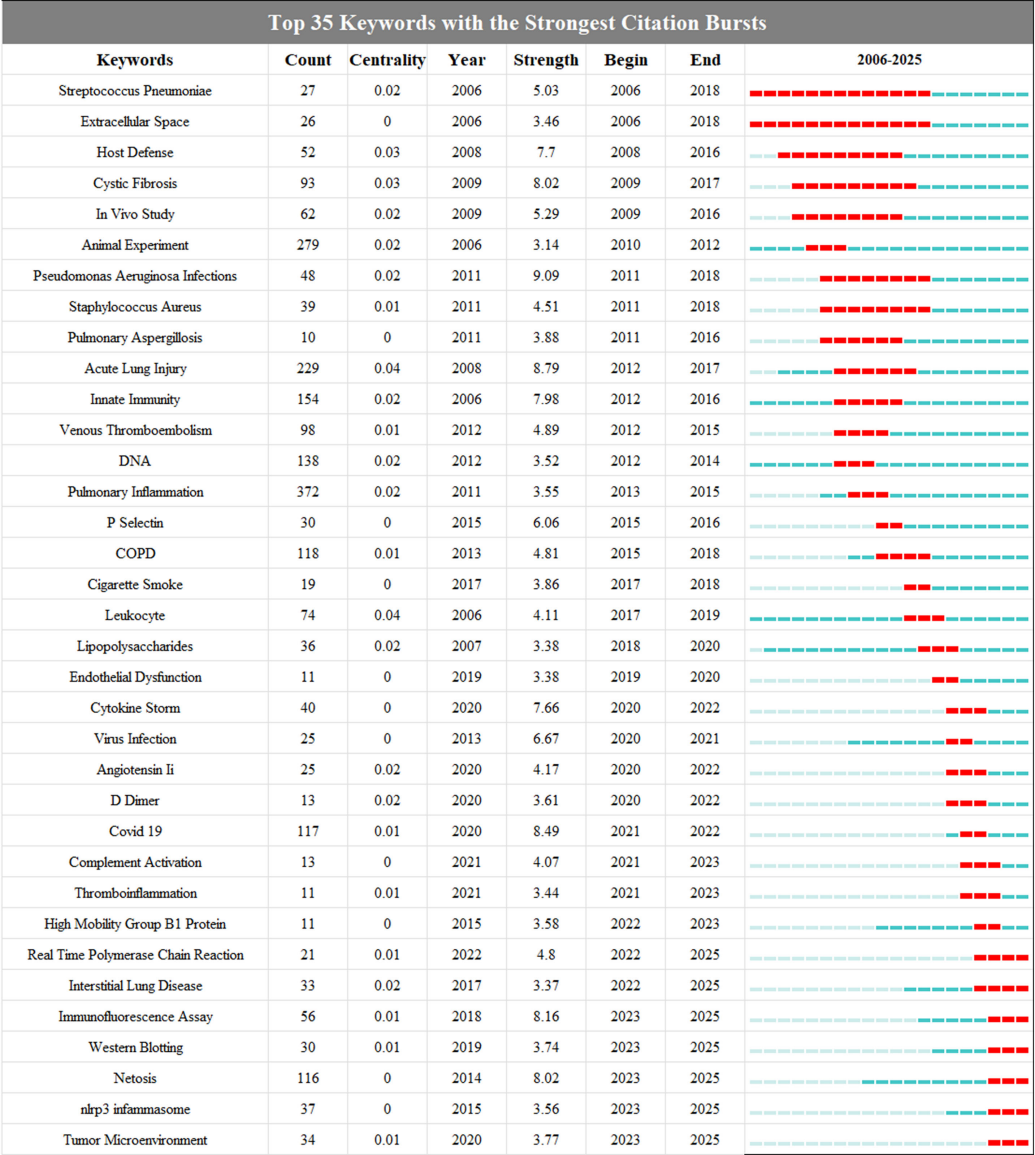
Top 35 keywords with the strongest burst.

**Figure 10** illustrates the 35 keywords with the highest burst intensity in this domain. The results indicate that “Pseudomonas Aeruginosa Infections” ranked first with a burst intensity of 9.09, and its burst state lasted for 8 years (2011–2018), indicating that the related research on NETs and Pseudomonas aeruginosa infection constituted the core hotspot of this domain during this phase. Additionally, the keywords “Streptococcus Pneumoniae” and “Extracellular Space” had the longest burst duration (2006–2018), indicating that the basic research directions related to this pathogen and the extracellular space have been continuously attracting attention over a longer period. In recent years, some keywords such as “ lipopolysaccharide” and “ cigarette smoke “ have had relatively lower annual burst intensities, indicating a decline in their phased attention. In contrast, the keywords “nlrp3 inflammasome”, “interstitial lung disease”, and “tumor microenvironment” maintained their citation burst until 2025, indicating that these directions still maintain high academic vitality to this day and represent emerging research frontiers in the current and future phase.

## 4 Discussion

This study thoroughly analysed research output, cross-regional collaboration, and essential advancements in the domain of research concerning NETs and pulmonary diseases, revealing a prominent tendency of growth and evolution in knowledge structure. This study will integrate quantitative analytical results to examine elements such as research status, hotspot evolution, and future trends, systematically elucidating the development sequence, emerging frontiers, and academic impact of this domain.

### 4.1 Research status of NETs in the domain of pulmonary diseases

#### 4.1.1 Analysis of the development trend of publications

Between 2006 and 2009, the research domain concerning NETs and pulmonary disease remained in its initial exploration phase. Mainstream research during this period concentrated on elucidating the creation mechanisms, immunological functions, and detection methods of NETs in pulmonary diseases such as LRTI and CF. The majority of the accomplishments remained confined to the phenomenal observation and descriptive analysis. From 2010 to 2018, the annual publication volume in this domain showed a stable growth trajectory, with the in-depth study of immunological mechanisms having elucidated the fundamental pathways of NET formation, including PAD4-mediated histone citrullination (19) and NADPH oxidase-independent NETosis (20), this advancement has transitioned research from mere phenomenal description to functional validation, leading to a growing acknowledgment of the “double-edged sword” characteristic of NETs in lung immune defense and pathological damage (21). After 2019, the annual publication volume reached a prominent phase of development, particularly exhibiting a marked growth inflection point in 2019. This trend is ascribed to the progressions of pertinent technology and the transformation of research backgrounds (22).

At the level of technological progressions, the proliferation of big data and cloud computing technologies has facilitated researchers in the efficient storage of extensive datasets, consequently promoting more teams to increase participation in clinical and fundamental translational research (23, 24). Simultaneously, progressions in methodologies, including multiplex immunofluorescence imaging (25), enzyme-linked immunosorbent assay (26), and western blotting (27), have accurately delineated the dynamic characteristics of NETs. In terms of research backgrounds, the COVID-19 pandemic has stimulated the global scientific community’s demand for studying the core role of NETs in the immunopathology of COVID-19, directly driving a surge in the initiation of related research projects and the publication of articles, it has also greatly promoted the cross-integration of multiple disciplines such as immunology, respiratory medicine, hematology, and bioinformatics, facilitating the analysis of the dual roles of NETs in the lung immune microenvironment from a systems biology perspective (28). Moreover, large-scale international research projects in China, the United States, Europe, and other nations have swiftly disseminated data and resources, expediting the validation of disciplinary hypotheses and the advancement of knowledge (29). The synergistic impact of these technological and environmental elements has resulted in a concentrated emergence of research progressions in this domain.

#### 4.1.2 Analysis of the geographical distribution pattern of publications

Ninety percent of the top 10 countries by worldwide publication volume are located in North America, Europe, and East Asia. With the exception of China and Brazil, all other countries are developed, indicating a prominent association between a country’s economic development and its academic output. This distribution characteristic aligns with the statistics on global biomedical research investment published by the WHO, indicating that high-income regions exhibit a markedly greater intensity of research expenditure for immune-related diseases; among them, North America constitutes 48% of worldwide R&D total expenditure, Europe 29%, and East Asia and the Pacific area 16% (30). This pattern indicates that the extent of fundamental research is predominantly constrained by the capacity to allocate research resources, and high-yield countries can enhance their academic contributions in this domain by sustained research investment and proactive policy support.

This pattern is especially prominent in the background of the COVID-19 pandemic. From 2019 to 2021, the publication volume of researchers from the majority of the top 10 countries peaked, and it was also the period with the fastest annual growth rate. Throughout this period, no public health event exerted a more significant global influence than the COVID-19 pandemic, particularly with the early surveillance and effective treatment of severe COVID-19 cases, which presented a substantial challenge. Research indicates that in severe COVID-19 cases, NETs are considered a major pathological factor contributing to immunological thrombosis and cytokine storm. NETs facilitate microvascular thrombosis by transporting tissue factor, activating complement C3/C5a and the coagulation cascade, while the histones and other mediators they release can intensify endothelial cell activation and damage to the alveolar barrier, thereby promoting the emergence of cytokine storm (31, 32). Furthermore, NETs serve as potential biomarker indicators that facilitate the early detection of high-risk individuals and the execution of clinical risk stratification treatment (33). Consequently, targeting the inhibition of NETs production or the clearance of existing NETs has emerged as a prominent area of research for mitigating thrombosis and inflammatory reactions in severe COVID-19 patients. Countries with high productivity in this domain usually possess traits such as extensive early exposure to the disease and swift scientific research emergency response systems. They can integrate a substantial quantity of clinical samples and research resources rapidly, consistently generating high-impact outcomes due to their robust research foundation, effective cross-institutional cooperation system, and patent conversion platform, thereby further consolidating their academic leadership (34). Undoubtedly, these research projects significantly contributed to addressing the pandemic and objectively reinforced the current global distribution pattern of research geography.

Notably, driven by the COVID-19 pandemic, China has emerged as the country Notably, driven by the COVID-19 pandemic, China has emerged as the country with the highest volume of publications in this domain (35). This tendency starkly contrasts with the results of a 2022 bibliometric analysis on NETs, which identified China as a rising region in the research domain of NETs and pulmonary diseases. This transition signifies China’s rapid advancement and substantial progress in this domain. Nonetheless, the United States and Germany surpass China in both the H-index and AC/P, indicating greater academic impact. This difference indicates that just tallying publications is inadequate for a thorough evaluation of the quality and influence of academic contributions; this may be attributable to various factors. On the one hand, some research exhibits deficiencies in experimental design rigor, comprehensive elucidation of NETs mechanisms, and validation of clinical correlations with pulmonary diseases, thus restricting their likelihood of citation in authoritative articles. On the other hand, a significant fraction of research achievements is published in domestic journals, which attract relatively little international attention and accessibility, hindering their inclusion and dissemination in mainstream international databases.

Overall, global academic achievements show notable geographical distribution characteristics; unless regional disaster events or systemic changes occur, it is challenging to diminish the academic influence and cooperative value derived from this geographical distribution. Factors including insufficient investment in scientific research infrastructure, the absence of interdisciplinary collaboration platforms, and the lack of strategic reserves for fundamental scientific research on sudden pulmonary diseases collectively hinder underdeveloped countries from attaining academic breakthroughs and establishing sustainable research systems in this domain. Consequently, these countries often struggle to make systematic progress in the research of NETs-related pulmonary disease mechanisms, the innovation of diagnostic technologies, and their translational application. However, as global comprehension of immune-mediated diseases advances and related technological platforms proliferate, transnational and cross-institutional cooperation networks are consistently expanding, offering potential avenues for resource-limited countries to engage in high-level research and facilitating their integration into the global scientific research system.

#### 4.1.3 Overview of research categories

In the current research domain, the subject categories of Immunology, Biochemistry & Molecular Biology, and Cell Biology have received relatively early systematic elaboration (**Supplementary Figure 1C**). Within the domain of Immunology, the most frequently cited article is “PAD4 is essential for antibacterial innate immunity mediated by neutrophil extracellular traps, “ with a total of 1, 169 citations. This study was the inaugural application of a PAD4 gene deletion mouse model to validate that PAD4 and the histone hypercitrullination it facilitates are essential prerequisites for NET formation (19). Within the domains of Biochemistry & Molecular Biology and Cell Biology, the most cited article is “Platelet TLR4 activates neutrophil extracellular traps to ensnare bacteria in septic blood, “ with a total of 1, 688 citations. This study elucidated the method by which platelets identify pathogens via TLR4 in septic circumstances, then activate neutrophils and trigger NET production, underscoring the immune defense function of NETs in effectively capturing bacteria in areas such as pulmonary capillaries. It provides a reliable theoretical framework for ongoing investigation of the role of NETs in pulmonary infections and associated inflammatory responses (36).

Subsequently, the research scope of NETs concerning pulmonary diseases has broadened to encompass several interdisciplinary domains, including Respiratory System, Pharmacology & Pharmacy, Medicine, Research & Experimental, Hematology, Microbiology, and Oncology. By 2019, this domain had attained profound integration with other high-frequency disciplinary categories. This cooperative strategy effectively promoted knowledge exchange and multidisciplinary innovation across several domains, consequently enhancing the theoretical advancement and practical application of NETs in pulmonary disease research. 2021 marked another significant milestone, as the volume of published articles across most pertinent research categories reached a peak (**Supplementary Figure 1C**). This was primarily due to the impetus of the COVID-19 pandemic. Undoubtedly, the worldwide pandemic outbreak compelled researchers to intensify their investigation into the potential therapeutic target of NETs for acute respiratory distress syndrome (ARDS) (31), while also substantially broadening research in domains such as pulmonary embolism (32), ALI (37), and other COVID-19 complications, thereby underscoring the significance of NETs as biomarkers and targets for therapeutic intervention.

In summary, a comprehensive and interdisciplinary medical platform has been established in the research domain concerning NETs and pulmonary diseases; the ongoing advancements in this domain are anticipated to enhance the innovation of clinical diagnosis and treatment for associated pulmonary diseases. Realizing this objective necessitates the meticulous cooperation of specialists and teams across various domains, including respiratory medicine, immunology, and bioinformatics. The significant value of NETs research is in the profound integration of interdisciplinary knowledge, which facilitates the elucidation of novel regulatory mechanisms of pulmonary diseases, fosters innovation in diagnostic and therapeutic tactics, and enhances patient prognosis.

### 4.2 Overview of knowledge maps and research hotspots

The research domain concerning NETs and pulmonary diseases has evolved from elucidating antibacterial mechanisms to differentiating their functional roles in various pulmonary diseases, including infections, thrombosis, chronic inflammation, malignancies, and COVID-19. The following section will outline the evolution of this domain in three phases: the initial exploration phase, the stable growth phase, and the significant development phase.

#### 4.2.1 Initial exploration phase (2006–2009)

From 2006 to 2009, the quantity of research concerning NETs and pulmonary diseases was comparatively scarce; representative achievements mostly centered on elucidating the molecular mechanisms of NETs as a type of programmed cell death and their function in host defense.

In 2007, Stephen R. Clark and colleagues initially established that TLR4 on platelet surfaces can stimulate platelet adhesion and activate neutrophils by recognizing TLR4 ligands in the bloodstream, thus facilitating the formation of NETs. The research further indicated that in the pulmonary capillaries of sepsis patients, this mechanism effectively captures bacteria, indicating that the formation of NETs mediated by platelet TLR4 is a unique antibacterial regulatory pathway in sepsis (36). In the same year, Tobias A. Fuchs and colleagues, utilizing a Streptococcus pneumoniae infection model, demonstrated that NETs may efficiently capture and eliminate pathogens *in vivo*, mostly depending on the barrier formed by DNA backbones and high concentrations of antibacterial proteins in the extracellular space. The two researches established the theoretical basis for NETs as an antibacterial immunological mechanism and supplied significant data for further detailed examination of their dual regulation and specificity in pulmonary diseases (38).

#### 4.2.2 Stable growth phase (2010 – 2018)

Between 2010 and 2018, the annual publication volume in this domain had an inverted hook-shaped trajectory, initially increasing and subsequently declining, indicating an accumulation of research momentum throughout this phase. During this phase, academic discourse became increasingly more specialized, with research emphasis directed towards specific disease kinds and extensive *in vitro* and *in vivo* experimental investigations conducted. At the same time, a succession of new-style targeted therapeutic medications has appeared; however, the associated drug safety concerns and ethical controversies have also garnered widespread attention within the academic community.

Between 2010 and 2011, the formation mechanism of NET was further elucidated. Pilsczek, Florian, and colleagues found that Staphylococcus aureus can trigger a rapid (5–60 minutes), reactive oxygen species (ROS) burst-independent and lysosome-pathway-independent production of NETs. This process entails the dissociation of the nuclear membrane and vesiculation, transporting NETs in intact vesicles to the extracellular space and then releasing them after rupture (39). Simultaneously, the correlation between NETs and immunity to certain pathogens began to be confirmed. It was not only confirmed that NETs have a direct killing effect on pathogens in pulmonary infections, including Pseudomonas aeruginosa pneumonia and pulmonary aspergillosis, and that restoring NETs’ function post-gene therapy for chronic granulomatous disease is crucial for reinstating antifungal defense (40–42). Nonetheless, during these phased discoveries were being made, there exists an underlying concern within the research over the dysregulation of NETs formation, which plays a dual function in lung tissue damage and immune defense. For instance, in cases of pneumonia and ALI caused by the influenza virus, excessive production of NETs can compromise the vascular endothelial barrier and worsen lung tissue damage (43); In the airways of CF patients, the abnormal accumulation of NETs can release a large amount of extracellular DNA and histones, which, after cross-linking with mucin glycoproteins, significantly increases the viscosity of sputum, thereby exacerbating airway obstruction, increasing clearance resistance, and promoting local inflammation and epithelial cell damage, ultimately leading to progressive decline in pulmonary function (44). The pathological issues resulting from NETs’ regulatory imbalance remain unresolved.

In 2012, the role of NETs in venous thrombosis emerged as a research hotspot in this domain. Numerous researches have established that NETs ensnare platelets and red blood cells via their distinctive DNA framework and histones, offering a binding surface for coagulation factors, thereby facilitating platelet aggregation and the coagulation cascade; concurrently, the released histones can effectively induce endothelial cell damage and apoptosis, intensifying the local prothrombotic environment (45–47). In the same year, Volker Brinkmann and his team, who identified NETs, systematically elucidated the biological characteristics and pathological implications of NETs in their review “Neutrophil extracellular traps: is immunity the second function of chromatin?”, highlighting that NETs are chromatin-protein complexes released via NETosis, with their formation contingent upon ROS, NE, MPO, and PAD4, they serve dual roles in innate immunity, encompassing pathogen capture and immune response activation, excessive production and clearance disorders may result in autoimmune, thrombotic, and inflammatory diseases (48). This article provided a comprehensive theoretical foundation for comprehending the function of NETs in diverse diseases; as of the time of this article’s search, it has been cited over 775 times.

Between 2013 and 2014, the research focus in this domain markedly transitioned to pulmonary inflammation. This phase of research expanded upon the prior comprehension of the function of NETs in infection and thrombosis, elucidating their dual significance in many inflammatory pulmonary diseases. A consensus gradually developed that the production of NETs and their components is not merely a byproduct of the inflammatory response but also a crucial factor in enhancing and extending the inflammatory response. In ALI and ARDS models, the production of NETs is closely associated with heightened pulmonary vascular permeability, damage to alveolar epithelial cells, and the cytokine storm, indicating that NETs may function as potential biomarkers for evaluating disease severity (49, 50). Especially in chronic airway inflammatory diseases such as BA, NETs can directly stimulate the proliferation of airway epithelial goblet cells and the expression of mucin protein MUC5AC, thereby exacerbating airway obstruction. They can also induce apoptosis of airway epithelial cells and damage ZO-1 and occludin, leading to loss of epithelial integrity and enhancing the permeability of allergens and pathogens. At the immunoregulatory level, the self-antigens and DNA complexes carried by NETs can activate plasmacytoid dendritic cells, promoting Th2 and Th17-type immune responses, thereby enhancing mixed airway inflammation involving eosinophils and neutrophils, and promoting the formation of hormone-insensitive asthma phenotypes (51). Furthermore, during this phase, research also began to systematically elucidate the key molecular mechanisms that regulate the formation of NETs, in addition to the established PAD4-dependent histone citrullination and ROS-dependent NETosis pathways, the calcium ion signaling pathway and the Raf-MEK-ERK signaling pathway significantly influence NET release, providing a theoretical foundation for future targeted therapeutic strategies against NETs (52).

Between 2015 and 2016, the research concentrated on the relationship between NETs and the pathological progression of LC; the primary objective was to investigate the function of NETs in constructing the inflammatory microenvironment and promoting tumor cell invasion and metastasis. Additionally, the research preliminarily assessed the associations between NETs-related markers, including H3Cit, MPO-DNA, and NE-DNA, in bronchoalveolar lavage fluid and sputum samples, alongside the severity and prognosis of LC patients (53). This research trend’s formation is ascribed to several factors. Firstly, the function of the TLR4-MAPK signaling pathway in the regulation of inflammation has become increasingly clear. NETs, as a crucial effector type mediated by neutrophils in inflammatory responses, their interaction with tumor cells has emerged as a pivotal focus for elucidating the mechanism by which inflammation promotes cancer (54). Secondly, conventional techniques for evaluating LC, including imaging examinations and tissue biopsies, have limits regarding sensitivity for early diagnosis and accuracy in prognostic prediction. This has driven researchers to investigate the auxiliary value of NETs-related markers in non-invasive diagnosis and stratified evaluation of LC (55). Furthermore, the previous research on the function of NETs in venous thromboembolism and pulmonary diseases like CF has offered both theoretical and technical foundations for its extended investigation in LC. Consequently, targeting NETs is gradually viewed as a potential strategy for LC therapy. Research indicates that inhibiting the PAD4 enzyme or using DNase I-coated nanoparticles to break down the DNA backbone of NETs can effectively obstruct NET-mediated tumor spread and the reactivation of dormant cancer cells (54, 56). Moreover, inhibitors aimed at key components of NETs, including NE and matrix metalloproteinase 9, or using antibodies to block the newly generated epitopes after NET-segment protease cleaves laminin, can disrupt the downstream oncogenic pathways of NETs, impede integrin activation, and hinder cancer cell invasion (57). These intervention strategies provide an experimental foundation for the advancement of novel combination treatments for LC.

In 2017, the publication volume in this domain surpassed 50 articles for the first time, about 27% of the research concentrated on the two primary inducing factors, cigarette smoke and lipopolysaccharide (LPS), which led to a dual perspective of environmental exposure and pathogen-mediated pathways. As a significant environmental factor, cigarette smoke primarily stimulates plasmacytoid dendritic cells, triggering a secondary inflammatory cascade and facilitating the formation of NETs (58). Research indicates that cigarette smoke can induce mitochondrial malfunction in neutrophils, impede their apoptotic process, and release DAMPs, thereby activating the innate immune response via receptors such as TLR2 and TLR4, while also enhancing NETosis. Furthermore, cigarette smoke can inhibit the histone deacetylase/Rac/CD9 signaling pathway, hindering the clearance of apoptotic neutrophils, resulting in the continuous release of NETs and their constituents, which further aggravates airway inflammation and tissue damage (58). It, along with respiratory irritants like flavored e-cigarette liquid, constitutes the main environmental exposure factor for NET-related lung injury, providing a key entry point for the research of diseases such as COPD and BA (59). On the other hand, LPS, a component of the cell wall of Gram-negative bacteria, serves as a classic pathogen-associated molecular pattern molecule for ALI and ARDS. LPS activates downstream signaling by interacting with the TLR4/MD2 complex, leading to the secretion of tumor necrosis factor-α and interleukin-8 (IL-8), which facilitates the recruitment and activation of neutrophils and enhances the excessive release of NETs via ROS-dependent or -independent pathways. This process is considered a core mechanism for the amplification of pulmonary inflammation and tissue injury (60). In recent years, intervention strategies targeting the LPS-NETs axis have proliferated, including mesenchymal stem cells, psoralen polysaccharides, and aspirin, which mitigate lung injury by modulating this axis; additionally (61–63), small-molecule compounds such as digitoflavone have demonstrated protective effects by inhibiting NET-related pathways (64).

In 2018, Genschmer et al. demonstrated that active neutrophils can release exosomes CD63+/CD66b+, which are rich in NE. The NE on the surface of these exosomes can effectively resist the suppression of α1AT and target the ECM via integrin Mac-1, resulting in significant lung tissue damage in COPD and bronchopulmonary dysplasia (65). This study elucidated a novel mechanism of NETs-associated damage and identified new targets for disease management and intervention.

#### 4.2.3 Significant development phase (2019 - present)

Between 2019 and 2022, the publication volume in the domain related to NETs and pulmonary diseases peaked, and it was also the phase during which the top 10 countries or institutions experienced the most rapid development in publication volume. The COVID-19 pandemic was the primary catalyst for this trend.

COVID-19 is induced by SARS-CoV-2 and has resulted in a worldwide pandemic, with over 270 million confirmed cases and roughly 5.3 million deaths. This disease can induce cytokine storm and multiple organ failure, which then progress to ARDS and a markedly elevated increased rate (66–68). Research indicates that NETs directly mediate pulmonary microvascular endothelial damage, immune thrombosis, and the amplification of cytokine storm, consequently advancing ARDS and its complications through the release of cytotoxic histones, pro-inflammatory mediators, and the activation of the coagulation cascade. In COVID-19 patients, the levels of key NET markers, including free DNA, MPO -DNA complexes, and citrullinated histone H3 in serum, significantly increased and were positively correlated with disease severity, particularly in patients necessitating mechanical ventilation. The levels of NETs were even higher (69, 70). The degradation therapy targeting NETs has emerged as a potential treatment strategy, the most widely discussed strategy is DNase therapy to rectify the impaired degradation of NETs in critically ill patients. Geoffrey Garcia et discovered an imbalance between excessive NET production and reduced endogenous DNase activity in individuals with severe COVID-19. The exogenous administration of recombinant rhDNase suppresses NETosis, reduces neutrophil infiltration and thrombosis, and improves respiratory symptoms and clinical prognosis (71). Currently, several clinical researches have confirmed it can improve patients’ respiratory symptoms and prognosis. Nonetheless, the uncertainty of clinical trial results and the necessity for enhancing drug specificity still necessitate empirical research to validate their clinical translational value, and further incorporate biomarkers to guide personalized treatment, thereby consolidating the academic status and clinical value of NETs in the treatment of COVID-19 and associated respiratory diseases.

Notably, the development of nanomedicine has provided a highly promising strategy for drug-targeted NETs therapy in ARDS induced by the SARS-CoV-2 virus. The PLGA-polypyrrole-polyethylene glycol composite nanoparticles can efficiently load and safeguard enzymes like DNase-1, markedly extending their half-life in the organism and enhancing their stability, thus constantly degrading cfDNA, inhibiting the NETosis process, mitigating cytokine storm, and minimizing lung tissue damage (72). Furthermore, nanoparticles can achieve targeted delivery via surface modification, increasing drug enrichment in pulmonary inflammation and improving therapeutic precision. However, this method still faces numerous challenges, including the swift elimination of nanoparticles by the mononuclear phagocytic system (73), potential toxicity from non-specific accumulation in the liver and kidneys (74), the intricacy of large-scale production (75), and immunogenicity concerns in clinical translation (76).

### 4.3 Overview of prospective trends

The keywords identified in the 2025 burst test signify the future research trends in the research domain related to NETs and pulmonary diseases post-2025, including “nlrp3 inflammasome”, “interstitial lung disease”, and “tumor microenvironment”, which represent the emerging research frontiers in both the present and future phase (**Figure 10**). The discussion will now concentrate on these three aspects.

#### 4.3.1 NETs and NLRP3 inflammasome

NETs and NLRP3 inflammasome, as pivotal effectors in the innate immune response, have become a cutting-edge focus in immunopathology owing to their interactive mechanisms in pulmonary diseases. The co-occurrence burst of the keywords “NLRP3 inflammasome” and “NETs” essentially reflects the clinical challenge posed by uncontrolled inflammatory cascades in pulmonary diseases. They jointly drive the amplification of inflammatory cascades and lung tissue damage through the establishment of a positive feedback loop. At the molecular level, constituents released by NETs, including histones, free DNA, and MPO, can activate the assembly of the NLRP3 inflammasome and promote the deubiquitination modification of the NLRP3 protein by causing a ROS burst, therefore markedly enhancing its activation efficiency (77). As Pierre-Andre Jarrot’s research team indicated that histone H3 derived from NETs can directly trigger the assembly of the NLRP3 inflammasome by activating the TLR4-MyD88 signaling pathway in macrophages, while ROS can promote the ASC oligomerization of the NLRP3 protein, thereby further enhancing inflammasome activation (78). Meanwhile, the activated NLRP3 inflammasome releases pro-inflammatory mediators, including IL-1β and IL-18, via the caspase-1-mediated pyroptosis pathway, which further recruits and activates neutrophils, promoting PAD4-dependent NET formation (79, 80). Moreover, cathepsin B participates in NETs formation by cleaving the gasdermin D protein, and mitochondrial DNA can directly activate NLRP3 in response to ROS stimulation, thereby exacerbating this vicious cycle (81, 82). In different pulmonary diseases, this interaction can present specific pathological manifestations. In ARDS, NETs intensify alterations in vascular permeability by forming microthrombi and directly damaging alveolar epithelial cells, while also activating the NLRP3 inflammasome in alveolar macrophages, inducing cell pyroptosis and the release of large inflammatory mediators, thereby promoting the occurrence of a cytokine storm (80). In COPD, persistent stimulation to cigarette smoke and other factors promotes NETs production via the TLR4/ROS dual pathway, NE and histones jointly exert cytotoxic effects, together with the continuously active NLRP3 inflammasome, sustain the chronic inflammatory microenvironment of the airway (83). At the onset of idiopathic pulmonary fibrosis (IPF), the NLRP3 protein can directly facilitate myofibroblast differentiation and collagen deposition independently of the classical inflammatory pathway, while components like MMP9 released by NETs promote the fibrotic process by disrupting extracellular matrix homeostasis (84). Given the central role of this vicious cycle in driving disease progression, intervention strategies targeting the NETs–NLRP3 axis demonstrate considerable promise. Plasma cell-free DNA, MPO-DNA complexes, and ASC oligomers can dynamically reflect the activation status of this axis, holding early warning value for cytokine storm in ARDS and acute exacerbation of IPF (85, 86); the potential of a colchicine plus low-dose aspirin combination regimen to reduce the frequency of acute exacerbations of COPD and to delay pulmonary function decline in IPF is being progressively validated (87–89). Current translational research is primarily focused on developing specific inhibitors against key nodes along this pathway, such as utilizing PAD4 inhibitors to inhibit NETs formation and employing NLRP3-specific inhibitors like MCC950 to disrupt inflammasome activation. Furthermore, the combined application of degradation therapy targeting NETs components and NLRP3 inhibitors can produce a synergistic protective effect in the LPS-induced acute lung injury(ALI) model, providing experimental evidence for the combined intervention strategy (90), and the priority of solving the local delivery problem of pulmonary aerosol inhalation preparations is a crucial step towards clinical application. In conclusion, achieving precise modulation of this pathway without damaging the host’s intrinsic defense mechanisms and further elucidating the context-specific mechanisms of the NETs-NLRP3 axis across different pulmonary diseases remains a critical challenge for future translational research. Breakthroughs in this domain are expected to establish novel paradigms for precision immunotherapy in inflammatory pulmonary diseases.

#### 4.3.2 NETs and ILD

ILD is a heterogeneous group of diseases characterized mostly defined by interstitial inflammation and fibrosis in the lungs. The pathological changes involve the destruction of the alveolar wall structure and aberrant deposition of extracellular matrix, severely hampering gas exchange functionality (91). Currently, the therapy of ILD mostly depends on glucocorticoids and immunosuppressants, but their efficacy is limited, particularly for patients with quickly progressing or fibrotic subtypes; the therapeutic effect is inadequate, and the prognosis is poor. Consequently, exploring the mechanisms of ILD and identifying new therapeutic targets has emerged as the main hotspot of contemporary research. NETs, as a key bridge between innate immunity and chronic inflammation and fibrosis, cause damage to pulmonary vascular endothelial cells and induce pyroptosis, while also promoting the transformation of lung fibroblasts into myofibroblasts through the activation of signaling pathways such as TLR9-miR-7-Smad2, thereby intensifying extracellular matrix deposition and the progression of lung fibrosis (92, 93). Jing Xue et al. observed an increase in NET markers, including Cit-H3, cfDNA, and MPO-DNA, in lung tissues and serum in models of experimental autoimmune myositis and rheumatoid arthritis-related ILD, and the levels of these markers were positively correlated with the severity of ILD (94). Furthermore, the manifestations of NETs differed among various ILD subtypes. In RA-ILD, MPO-DNA was significantly raised in the non-specific interstitial pneumonia subtype, but Cit-H3 was more significant in the UIP subtype, indicating that NETs are involved in the formation of different pathological phenotypes of ILD. In terms of clinical translation, the present research hotspot is on identifying NETs or other biomarkers that can signify the activity and prognosis of ILD diseases. The simultaneous detection of plasma MPO-DNA, Cit-H3, cfDNA, and autoantibodies such as RF and anti-CCP can improve the diagnostic precision for RA-ILD subtypes; the area under the ROC curve can exceed 0.85, demonstrating efficient clinical application potential (94). Future research needs to determine the clinically applicable cut-off values and evaluate whether this model can detect ILD earlier than high-resolution computed tomography in asymptomatic high-risk RA patients. On the other hand, treatment strategies targeted at NETs are currently in the exploratory phase. Research indicates that colchicine can reduce ILD and fibrosis in the EAM model mice by inhibiting microtubule polymerization, neutrophil chemotaxis, and NETs formation. Cl-aminolevulinic acid, as a PAD4 inhibitor, can also effectively block the release of NETs (95). These findings provide a theoretical basis and experimental support for a novel therapeutic strategy to ILD.

#### 4.3.3 NETs and TME

In the formation and accumulation of the tumor microenvironment (TME) in LC, NETs promote tumor progression, metastatic recurrence, and immune evasion by regulating immune responses, interfering with cell signal transduction, and engaging in matrix remodeling. Research indicates that tumor-derived IL-8 and Granulocyte Colony-Stimulating Factor, along with common hypoxic and high lactic acid states in the TME, strongly induce the formation of NETs (96, 97). Upon formation, NETs can obstruct the identification and elimination of tumor cells by cytotoxic T lymphocytes or natural killer cells due to their physical structure, while simultaneously activating the PI3K/AKT and NF-κB signaling pathways within cancer cells by transporting histones and NE (98, 99). NETs can also degrade T cell chemotactic factors via protease activity, recruit and activate regulatory T cells and myeloid-derived suppressor cells, collectively creating a highly immunosuppressive environment (99). Concurrently, NETs can facilitate tumor local infiltration and distant metastasis by degrading the ECM, releasing angiogenic factors including vascular endothelial growth factor, and directly capturing circulating tumor cells (100). Furthermore, as an important marker and amplifier of inflammation and oxidative stress within the TME, the ROS and proteases released during the formation of NETs can damage surrounding tissues, sustain a chronic inflammatory condition, and release more tumor-associated antigens and damage-associated molecular patterns, thereby continuously activating TLRs for innate immune signaling, forming a positive feedback loop that promotes tumor development (101, 102). Considering the various functions of NETs within the TEM in LC, targeting NETs has emerged as a potential therapeutic strategy. In preclinical models, local administration of DNase I to degrade NETs combined with anti-PD-1 therapy restores T-cell-mediated cytotoxicity against tumor cells. For postoperative or post-chemoradiotherapy patients who are ctDNA-positive but show no radiographically detectable lesions, low-dose PAD4 inhibitors or sivelestat are currently under experimental validation for preventing distant metastasis driven by minimal residual disease. Furthermore, combinatorial analytical models that integrate NET remnant markers in plasma, such as citrullinated histones, with tumor mutational burden or ctDNA mutant allele frequency have demonstrated potential for early warning of immune evasion or epithelial–mesenchymal transition in early screening and therapeutic monitoring of LC (103–105). In conclusion, strategies targeting NETs in combination with existing chemoradiotherapy or immunotherapy, along with the precise development of NET-associated biomarkers, are expected to ultimately improve the prognosis of LC patients and overcome therapeutic resistance. Future research should further elucidate the functional heterogeneity of NETs at different stages of LC, optimize combination regimens of NET-targeted therapy with chemoradiotherapy and immunotherapy, and develop precise NET-related biomarkers and therapeutic targets. Through rigorous preclinical and clinical validation, NET-targeting strategies hold promise to be translated into effective clinical interventions that improve prognosis and overcome therapeutic resistance in patients with LC.

## 5 Limitations

This study is the inaugural application of bibliometric tools and visualization techniques for a systematic analysis of the developmental and evolutionary trends in the domain of research concerning NETs and pulmonary diseases over the past 20 years. However, this study still has certain limitations. Firstly, the article’s sources are limited to the WoSCC and Scopus databases, which may result in the omission of authoritative studies from databases such as PubMed and Embase. Secondly, this study only covers English-language articles and excludes research results published in other languages, which may introduce language bias. This limitation may result in some high-quality studies published in non-English countries in their native languages being excluded, thereby potentially underestimating their academic contributions to a certain extent. Furthermore, due to the influence of the update timeliness of the databases, the articles published after the retrieval deadline of December 31st, 2025, were excluded, resulting in a certain temporal bias. Simultaneously, in citation analysis, the time lag effect in citation accumulation may lead to an underestimation of the academic influence of recently published articles, so affecting the completeness and objectivity of the research results.

## 6 Conclusion

Based on the bibliometric analysis of NETs and pulmonary diseases, this study found that this domain is receiving increasingly widespread attention from the global academic community. According to the annual publication trend, the related research on NETs and pulmonary diseases showed an initial exploration and stable growth from 2006 to 2018, then experienced significant development driven by the COVID-19 pandemic after 2019, reaching a peak in 2022. Based on this, it can be inferred that this research direction will continue to maintain an active development trend in the future. In terms of the scale of publications and geographical contributions, the United States, China, and Germany are the core countries. China has the highest number of publications, but the United States maintains a leading position in academic influence and high-quality research areas with a high H-index, BC, and AC/P. Through a comprehensive assessment of publication and citation data, it is indicated that journals such as “Frontiers in Immunology” and “International Journal of Molecular Sciences “ that focus on the intersection of immunology and pulmonary diseases have become the core journals for research results in this domain. Through reference literature clustering, keyword co-occurrence, and emergent graphs analysis, the hotspots of the domain were further identified. From the above hot topics, it can be known that the current research focus has concentrated on the dual regulatory role of NETs in the occurrence and progression of pulmonary diseases; and these researches around the regulatory role have gradually clarified the direction of targeted intervention on NETs to improve the pathological process of pulmonary diseases, with the aim of serving as a reference strategy for many scholars to break through the therapy bottleneck of pulmonary diseases.

## Data Availability

The raw data supporting the conclusions of this article will be made available by the authors, without undue reservation.
