# Supplementary material for: From immunological mechanisms to targeted therapies: a bibliometric analysis in the domain of research concerning neutrophil extracellular traps and pulmonary diseases (2006–2025)
Source: Front Immunol. 2026 Jun 3;17:1837492. doi: 10.3389/fimmu.2026.1837492 (PMC13271971; doi:10.3389/fimmu.2026.1837492)
Supplement: Supplementary file 1 [file DataSheet1.docx]

| **Pesearch protocol** | **Retrieve results and contents** |
| --- | --- |
| Research database | WoSCC(SSCI SCI) And Scopus |
| Retrieval criterion | TS=((“Extracellular Trap*” OR “Extracellular DNA Trap*”) AND (“Lung” OR “Pulmonary”)) |
| Year span | January 1st, 2006—December 31st, 2025 |
| Search date | December 31st, 2025 |
| Initial text volume | WoSCC（n=2009）Scopus（n=2445） |

**Supplementary Table 1**


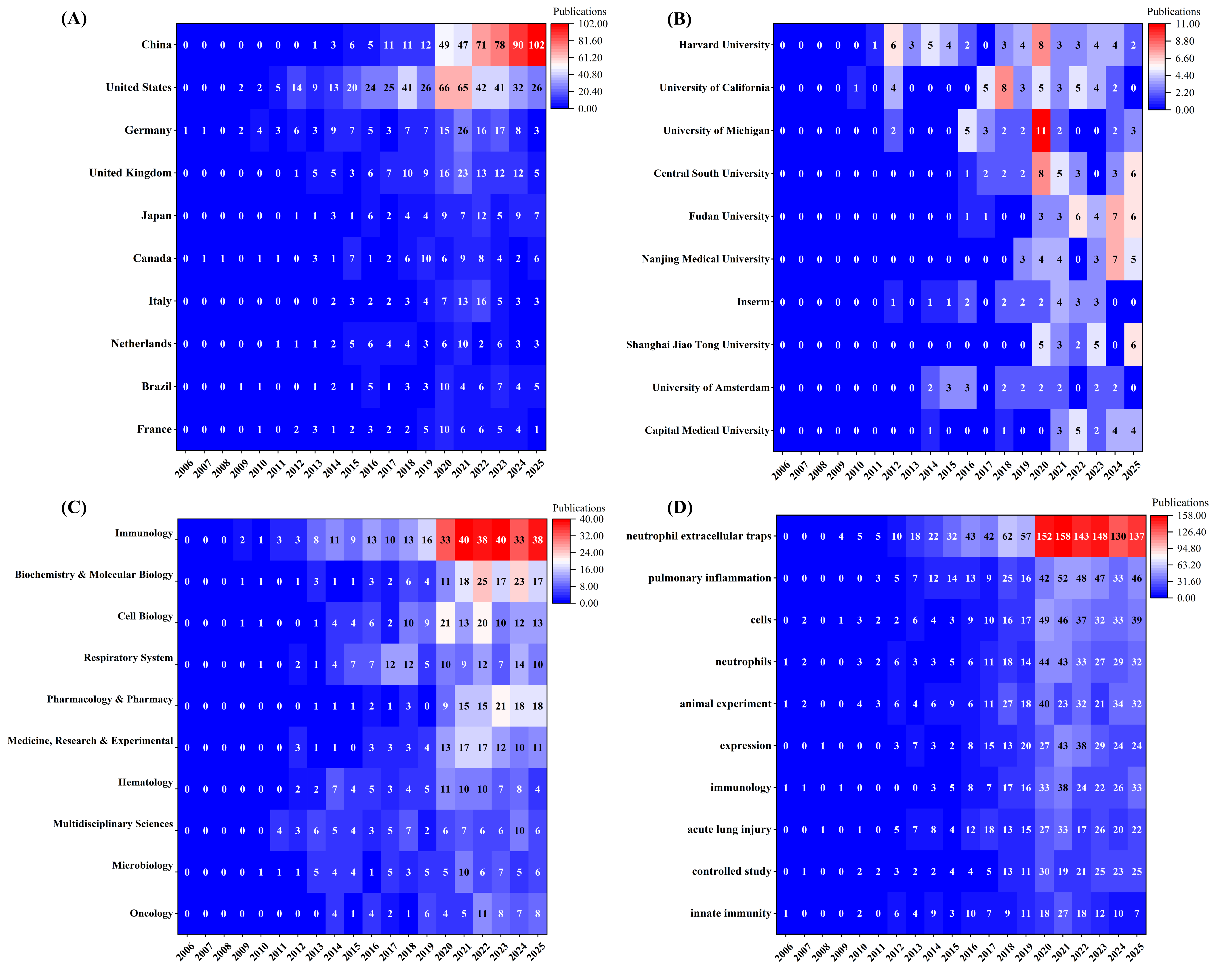


**Supplementary Figure 1 (A - D) Annual fluctuations in the frequency of NETs in the domain of pulmonary diseases among high-yield countries/regions (top 10), high-yield institutions (top 10), frequently studied categories (top 10), and frequently used keywords (top 10).**
